# Does Auricular Acupoint‐Related Therapy Enhance Rehabilitation After Total Knee Arthroplasty? A Systematic Review and Network Meta‐Analysis

**DOI:** 10.1155/prm/9277186

**Published:** 2026-09-20

**Authors:** Qindong Mi, Yunxi Xu, Fei Bao, Xiaolong Zhao, Lijin Liu, Zhihong Wu

**Affiliations:** ^1^ Department of TCM, Peking Union Medical College Hospital, Chinese Academy of Medical Sciences & Peking Union Medical College, Beijing 100730, China, cams.ac.cn; ^2^ Department of Ophthalmology, Hospital of Chengdu University of Traditional Chinese Medicine, Chengdu 610072, Sichuan, China, scu.edu.cn; ^3^ Department of Orthopaedic Surgery, Peking Union Medical College Hospital, Chinese Academy of Medical Sciences & Peking Union Medical College, Beijing 100730, China, pumch.cn; ^4^ Stem Cell Facility, Institute of Clinical Medicine, Peking Union Medical College Hospital, Chinese Academy of Medical Sciences & Peking Union Medical College, Beijing 100730, China, cams.ac.cn; ^5^ State Key Laboratory of Complex Severe and Rare Diseases, Peking Union Medical College Hospital, Chinese Academy of Medical Sciences & Peking Union Medical College, Beijing 100730, China, cams.ac.cn

**Keywords:** auricular acupoint-related therapy, functional recovery, pain management, postoperative rehabilitation, total knee arthroplasty

## Abstract

**Background:**

Although auricular acupoint‐related therapy (AART) is increasingly used for postoperative rehabilitation after total knee arthroplasty (TKA), evidence comparing the effectiveness of different AART modalities remains limited. This study aimed to evaluate the efficacy and safety of AART through a systematic review and network meta‐analysis (NMA).

**Methods:**

We performed a systematic review and NMA of randomized controlled trials (RCTs) from multiple Chinese and English databases, from inception to June 2026. Two reviewers independently screened studies, extracted data, and assessed risk of bias using the Cochrane ROB‐2 tool and evidence quality with GRADE. Pairwise comparisons and NMA were conducted using RevMan 5.4 and R software.

**Results:**

Twenty‐four RCTs involving 1790 patients were included. Compared with routine rehabilitation, AART significantly reduced resting pain scores on postoperative day 1 (mean difference [MD] = −0.38) and day 3 (MD = −0.61) and improved movement‐evoked pain on days 3 and 7. AART also enhanced knee function (Hospital for Special Surgery scores), increased active range of motion, and reduced complications such as vomiting, dizziness, and urinary retention. NMA suggested that auricular acupressure combined with Chinese medicine transdermal therapy may be the most effective intervention for early resting pain. Of note, the certainty of evidence for most outcomes was low or very low, and the findings should be interpreted with caution in clinical practice, serving only as preliminary reference rather than definitive clinical guidance.

**Conclusion:**

AART may serve as an effective non‐pharmacological adjunct in multimodal rehabilitation after TKA, particularly for acute pain relief, functional recovery, and complication reduction. High‐quality trials are needed to confirm these findings.

## 1. Introduction

Total knee arthroplasty (TKA) is a common and effective treatment for end‐stage knee osteoarthritis, significantly improving long‐term joint function and quality of life [1, 2]. However, postoperative management remains challenging. As a major orthopedic procedure, TKA is often accompanied by pain [3], lower limb swelling [4], impaired quadriceps strength, gait abnormalities, an increased risk of falls [5], and other adverse effects [6]. These complications can delay recovery and negatively affect surgical outcomes, with 16%–20% of patients reporting dissatisfaction with their rehabilitation results [6, 7]. Traditionally, postoperative pain management relies heavily on opioids [8]. Increasing awareness of their side effects has shifted clinical practice toward multimodal analgesia and comprehensive rehabilitation protocols, including acupuncture [9], aiming to enhance therapeutic efficacy while minimizing drug‐related risks.

Auricular acupoint therapy is a technique that stimulates specific auricular points using specialized instruments for disease prevention and treatment [10]. Auricular acupoint‐related therapy (AART) has shown potential in postoperative pain management [11]. In recent years, with a growing number of randomized controlled trials (RCTs), various AART interventions such as auricular acupressure (AP), auricular intradermal needling (AIDN), and transcutaneous auricular vagus nerve stimulation (Ta‐VNS) have been applied to enhance rehabilitation outcomes after TKA [12–14]. Although previous meta‐analyses suggest that AART can reduce postoperative pain in TKA patients [15], they have limitations. First, AART includes diverse treatment protocols, and existing reviews often lack subgroup analyses to differentiate specific intervention types. Second, they do not fully compare the efficacy and safety of different AART approaches or assess the quality of evidence. As a result, it remains unclear which auricular intervention offers the best balance of efficacy and safety for postoperative TKA rehabilitation. To address these gaps, we conducted a systematic review and Bayesian network meta‐analysis (NMA) of RCTs following Cochrane methodology. This study aims to comprehensively evaluate the effects of different AART regimens on postoperative outcomes after TKA and provide robust evidence to guide clinical decision‐making.

## 2. Methods

This systematic review was conducted following Cochrane meta‐analysis methodology [16] and reported according to the PRISMA 2020 checklist [17]. The study protocol was prospectively registered with PROSPERO (CRD420251030062). An amendment was made on 21 October 2025 to include an NMA before data synthesis.

## 3. Search Strategy

A comprehensive search was conducted using both database retrieval and supplementary methods. The databases included SinoMed, Embase, Web of Science, Cochrane Library, PubMed, CNKI, VIP, and Wanfang. Searches covered each database from inception to June 30, 2026. The strategy combined subject headings with free‐text terms, with detailed search terms provided in Supporting Information 1. Additional methods included author and citation tracking.

### 3.1. Eligibility Criteria

#### 3.1.1. Inclusion Criteria

Study Design: RCTs. Participants: Patients diagnosed with unilateral primary osteoarthritis who underwent TKA. No restrictions were applied for age, sex, or geographical region. Interventions: Experimental interventions had to involve AART, including but not limited to AP, auricular acupuncture (AA), AIDN, Ta‐VNS, and combination therapies (CTs). Control interventions were unrestricted. Outcomes: Studies must report at least one relevant outcome on patient function, pain, adverse reactions, or the safety profile of AART.

#### 3.1.2. Exclusion Criteria

Methodological Deficiencies: Studies that merely mention “randomized” without describing the randomization method, or those with clear flaws in the randomization process. Ineligible Publication Types: Duplicate publications, animal studies, theoretical papers, and dissertations. Irrelevant Study Scope: Studies focusing on bilateral TKA, unicompartmental knee arthroplasty, or other unrelated surgical procedures. Unavailable Data: Studies with missing original data that cannot be reasonably estimated through statistical methods, even after contacting the authors.

### 3.2. Data Collection Process

Literature screening was independently conducted by two researchers using EndNote 20. Each reviewer sequentially examined titles, abstracts, and full texts of the retrieved records to identify studies that met the inclusion criteria. After the initial screening, the two reviewers cross‐checked their results. Any discrepancies were resolved by a third‐party expert to reach a final consensus on study inclusion. Data extracted included author names, publication year, patient demographics (e.g., age and sex), and primary outcome measures. Relevant baseline data and mean values with standard deviations (SDs) for outcomes at different postoperative time points were also collected. When essential data were not available in the main text or public databases, the corresponding authors were contacted via email. If no response was received after two attempts within one month, the data were considered unavailable.

### 3.3. Risk of Bias Assessment

Two reviewers independently assessed the methodological quality and risk of bias of the included studies using the Cochrane risk of bias tool (ROB‐2) [18]. Each reviewer conducted the assessment individually and then cross‐checked their judgments. Any discrepancies were resolved by consensus or through consultation with a third researcher.

### 3.4. Statistical Analysis

Data analysis was performed using RevMan 5.4.0 and R 4.5.0. Forest plots and funnel plots were generated as appropriate. Sensitivity analysis was conducted using the leave‐one‐out method with the metafor package in R, and publication bias was assessed using Egger’s test when more than 10 studies were included. Because recovery after TKA is a dynamic process, outcomes were analyzed at multiple postoperative time points. Data were either directly extracted from the literature or calculated as needed. For partially missing SDs, estimation methods recommended by the Cochrane Handbook (https://training.cochrane.org/handbook) were applied. Heterogeneity was assessed using the I^2^ statistic. An I^2^ value > 50% indicated substantial heterogeneity, in which case a random‐effects model was used; otherwise, a fixed‐effects model was applied. In cases of considerable clinical heterogeneity, the random‐effects model was used regardless of the I^2^ value.

To compare the relative effectiveness of different AART regimens after TKA, we conducted a Bayesian NMA, which integrates direct and indirect evidence within a single statistical model. Only intervention subgroups with at least five studies were included to ensure the reliability of the NMA. A random‐effects model was consistently applied to account for potential clinical heterogeneity across interventions. The analysis was performed using the multinma package in R (version 4.5.0), with statistical significance determined by 95% credible intervals (CrIs). The network structure was visualized using the routine rehabilitation treatment (RRT) group as the common reference. Model consistency was evaluated both locally and globally: the node‐splitting method assessed local inconsistency, while the Deviance Information Criterion (DIC) was compared between consistency and unrelated mean effects (UME) models to evaluate global inconsistency. A ΔDIC > 5 was considered indicative of significant inconsistency. Finally, treatment hierarchies were generated using the Surface Under the Cumulative Ranking Curve (SUCRA) derived from the NMA.

### 3.5. Certainty of Evidence

First, the GRADE Profiler (version 3.6) was used to assess the quality of evidence. The GRADE approach provides a systematic framework for evaluating evidence and developing clinical recommendations [19]. Two reviewers then independently rated the evidence from the updated meta‐analyses using this framework. Final consensus on evidence quality was reached through discussion.

## 4. Results

### 4.1. Screening

**FIGURE 1:**
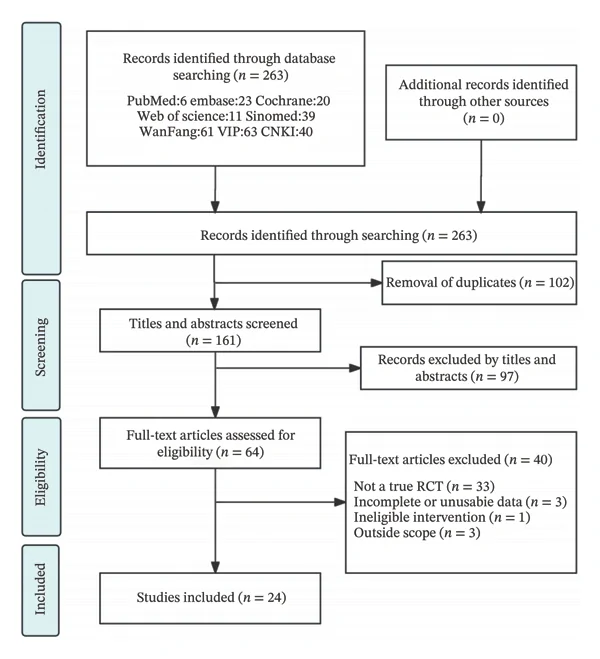
Flow diagram of the study selection process.

A total of 263 potentially relevant publications were identified through a systematic search, from which 24 RCTs [12–14, 20–40] met the eligibility criteria and were included in the final analysis. The literature selection process is detailed in Figure 1.

### 4.2. Study Characteristics

**FIGURE 2:**
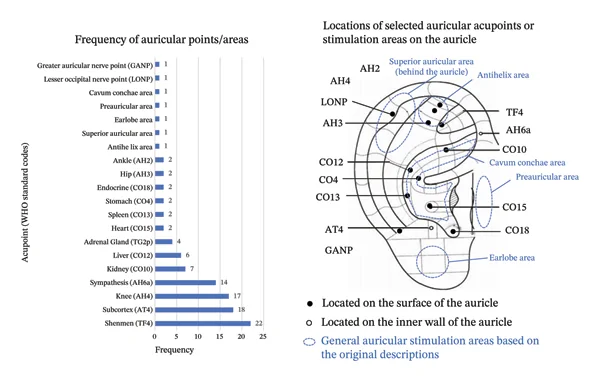
Frequency of auricular point/area utilization across the included studies.

All included studies enrolled patients who had undergone TKA, as defined in the Methods section, with a total of 1790 participants. The demographic characteristics and basic study features of the included RCTs are summarized in Tables 1 and 2, respectively. Detailed descriptions and definitions of all complementary therapies are provided in Supporting Information 1: Overview of Complementary Therapy Interventions. Figure 2 illustrates the locations of all auricular points identified in the included studies and reports their frequency of use. The analysis identified Shenmen, Subcortex, Knee, and Sympathetic as the most predominantly utilized points.

**TABLE 1:** Characteristics of all the participants of the included studies.

References	Region	Sample size		Gender (male, female)		Age (years)		BMI (kg/m^2^)		Symptom duration (years)		Operative time (minutes)	
		A	C	A	C	A	C	A	C	A	C	A	C
Ilfeld 2025	Mainland, USA	15	15	(9, 6)	(7, 8)	70.0 ± 7.0	68.0 ± 12.0	30.0 ± 5.0	29.0 ± 5.0	NA	NA	115.0 ± 22.0	109.0 ± 14.0
Chen 2025	Mainland, China	33	33	(8, 25)	(13, 20)	55.3 ± 8.9	54.6 ± 8.8	NA	NA	5.5 ± 1.9	5.5 ± 1.9	NA	NA
Zhang 2024	Mainland, China	48	48	(20, 28)	(9, 39)	67.9 ± 6.6	66.5 ± 7.5	27.0 ± 5.1	28.0 ± 5.1	10.5 (6.6, 15.0)	10.8 (7.3, 16.0)	NA	NA
Huang 2023	Mainland, China	56	56	(19, 37)	(20, 36)	71.6 ± 5.5	71.4 ± 5.7	24.6 ± 1.2	24.45 ± 1.2	NA	NA	NA	NA
Liu S 2023	Mainland, China	35	33	(14, 21)	(14, 19)	63.7 ± 4.7	63.5 ± 4.4	26.0 ± 2.2	27.0 ± 2.3	4 (2.0, 6.3)	5 (3.5, 8.0)	69.08 ± 16.78	62.51 ± 12.92
Liu X 2023	Mainland, China	40	40	(13, 27)	(16, 24)	61.5 ± 1.2	61.3 ± 1.2	NA	NA	(4.00, 7.00)	(4.0, 8.0)	NA	NA
Yang 2023	Mainland, China	30	30	(8, 22)	(7, 23)	70.6 ± 6.8	71.2 ± 6.6	NA	NA	NA	NA	NA	NA
Zhong 2023	Mainland, China	50	50	NA	NA	65.8 ± 1.4	64.6 ± 0.7	20.5 ± 3.0	20.77 ± 0.3	NA	NA	102.05 ± 2.121	104.70 ± 1.414
Wan 2022	Mainland, China	43	43	(24, 19)	(22, 21)	54.4 ± 4.3	54.2 ± 4.3	23.71 ± 1.3	23.67 ± 1.3	NA	NA	NA	NA
Wan 2022a	Mainland, China	46	46	(20, 26)	(25, 21)	65.4 ± 5.5	65.3 ± 5.4	23.0 ± 1.0	23.06 ± 1.0	NA	NA	NA	NA
Xiao 2022	Mainland, China	56	56	(7, 49)	(10, 46)	70.0 ± 9.1	71.0 ± 10.1	NA	NA	7.60 ± 1.09	7.64 ± 1.08	NA	NA
Sun 2021	Mainland, China	68	68	(26, 42)	(29, 39)	66.9 ± 5.2	67.2 ± 5.2	NA	NA	NA	NA	NA	NA
Liao 2020	Mainland, China	20	20	(2, 18)	(5, 15)	65.2 ± 10.9	65.0 ± 11.2	NA	NA	NA	NA	NA	NA
Sun 2020	Mainland, China	20	20/20	(9, 11)	(12, 8)/(10, 10)	79.5 ± 2.8	80.5 ± 2.7/76.5 ± 2.8	NA	NA	2.13 ± 0.23	2.12 ± 0.24/2.16 ± 0.21	NA	NA
Du 2020	Mainland, China	30	30	(7, 23)	(6, 24)	65.0 ± 3.0	64.4 ± 2.8	NA	NA	NA	NA	NA	NA
Si 2017	Mainland, China	20	20	(11, 9)	(14, 6)	65.5 ± 6.1	64.7 ± 6.7	NA	NA	NA	NA	134.9 ± 22.4	129.7 ± 23.5
Chen 2015	Taiwan, China	30	30	(3, 27)	(8, 22)	68.9 ± 9.0	69.0 ± 8.6	NA	NA	NA	NA	83.0 ± 25.0	79.0 ± 33.0
Tian 2015	Mainland, China	45	45	(27, 18)	(29, 16)	61.9 ± 7.6	62.2 ± 8.5	NA	NA	NA	NA	100.8 ± 15.2	104.2 ± 18.5
Wei 2015	Mainland, China	35	35	(19, 16)	(18, 17)	65.0 ± 7.1	64.0 ± 6.4	NA	NA	NA	NA	NA	NA
Zhang 2014	Mainland, China	30	30	(27, 18)	(29, 16)	65.8 ± 6.7	64.4 ± 7.9	NA	NA	NA	NA	NA	NA
He 2013	Mainland, China	45	45	(18, 27)	(16, 29)	62.6 ± 6.1	61.6 ± 6.7	NA	NA	NA	NA	NA	NA
Tian 2013	Mainland, China	30	30	(13, 17)	(11, 19)	64.2 ± 6.9	62.4 ± 8.1	NA	NA	NA	NA	NA	NA
Chang 2012	Taiwan, China	31	31	(3, 28)	(6, 25)	71.2 ± 7.1	70.7 ± 8.1	NA	NA	NA	NA	NA	NA
Tong 2010	Mainland, China	30	30	(11, 19)	(12, 18)	70.0 ± 4.8	71.4 ± 3.2	NA	NA	NA	NA	NA	NA

Abbreviation: NA, not available.

**TABLE 2:** Main features of all the included studies.

References	Intervention					Outcome	Adverse events		
	Type based on routine rehabilitation treatment		Auricular points/areas	Treatment period	Dosage and frequency	Indicator	Type	A	C
	A	C							
Ilfeld 2025	Ta‐VNS	Sham Ta‐VNS	Antihelix, Superior Auricular Region, Earlobe, Preauricular Area	POD‐1 ∼ POD 3 (5 days)	Unilat, Continuous Stimulation	②⑧	AAR	3	0
Chen 2025	Ta‐VNS		Cavum Conchae	POD 1 ∼ POD 7 (7 days)	2 Hz DDW, 2–6 mA, 20 min/sess, unilat, BID	③⑧/SAS	POPC	3	4
Zhang 2024	AIDN		Shenmen (TF4), Adrenal (TG2p), Knee (AH4), Subcortex (AT4), Stomach (CO4)	POD‐1 ∼ POD 3 (5 days)	Continuous stimulation, unilat, postop day 1: surgical side, postop days 1–3: contralateral side	①②④⑦⑨	AAR	0	0
Huang 2023	Ta‐VNS + AP		Shenmen (TF4), Sympathetic (AH6a), Liver (CO12), Kidney (CO10), Knee (AH4)	POD‐1 ∼ POD 7 (9 days)	Press 2–3 min/point, unilat, TID‐QID, PRN, replace seeds q3d, alternate ears	①②③⑨/TFA/DDP/PLOS	POPC	5	13
Liu S 2023	AP		Shenmen (TF4), Sympathetic (AH6a), Subcortex (AT4), Kidney (CO10), Knee (AH4)	POD‐1 ∼ POD 3 (5 days)	Press 1 min/point, unilat, TID, replace seeds q2d, alt ears	①②⑤	NA		
Liu X 2023	AP		Knee (AH4), Kidney (CO10), Liver (CO12), Shenmen (TF4), Sympathetic (AH6a)	POD 0 ∼ POD8 (9 days)	Unilat, TID, replace seeds q3d, alt ears	①④⑨	AAR	1	0
Yang 2023	AP		Liver (CO12), Kidney (CO10), Shenmen (TF4), Adrenal (TG2p), Knee (AH4), Lesser Occipital Nerve Point, Greater Auricular Nerve Point	POD‐3 ∼ POD 14 (18 days)	Press 1‐2 min/point TID, replace seeds q3d alt ears	①⑤/McGill/PPI	NA		
Zhong 2023	AP	Sham AP	Knee (AH4), Kidney (CO10), Shenmen (TF4), Subcortex (AT4)	POD 0 ∼ POD 3 (4 days)	Press 2 min/point, unilat, QID, replace seeds q3d alt ears	①⑤/Hb/PDV	NA		
Wan 2022	AP + CMTT		Sympathetic (AH6a), Shenmen (TF4), Subcortex (AT4), Knee (AH4)	POD 0 ∼ POD8 (9 days)	Press q30 min, 2 min/point, bilat, replace seeds q2‐3d bilat ears	①⑤⑥/PSQI	NA		
Wan 2022a	AP + CMTT		Sympathetic (AH6a), Shenmen (TF4), Subcortex (AT4), Knee (AH4)	POD 0 ∼ POD3 (4 days)	Press 3–5 min/point, BID, PRN, and replace seeds q2d	①④⑩	NA		
Xiao 2022	AP + Mox		Shenmen (TF4), Heart (CO15), Subcortex (AT4).	POD 0 ∼ POD3‐5 (4–6 days)	Unilat, alt ears	①④⑥	NA		
Sun 2021	AP + Massage		Heart (CO15), Liver (CO12), Spleen (CO13), Knee (AH4), Hip (AH3), Ankle (AH2).	POD 1 ∼ POD14 (14 days)	Press 2–3 min/point, unilat, TID‐QID	⑥/DVT	POPC	2	9
Liao 2020	AIDN		Shenmen (TF4), Knee Joint (AH4), Sympathetic (AH6a), Subcortex (AT4).	POD 1 ∼ POD3 (3 days)	Press 1 min/point, surgical side only, QID; replace seeds q3d ears	①②⑨/TFF/TFBM	POPC	3	9
Sun 2020	AP + CMTT	CMTT/AP	Shenmen (TF4), Sympathetic (AH6a), Subcortex (AT4), Knee (AH4)	POD‐1 ∼ POD 6–9 (8–11 days)	1 min/point, PRN, Preop: q2h × 3, Postop: q30 min Press, bilat, replace seeds q2‐3d bilat ears	①②⑤/McGill	NA		
Du 2020	AP		Shenmen (TF4), Sympathetic (AH6a), Subcortex (AT4), Kidney (CO10), Knee (AH4), Spleen (CO13), Stomach (CO4)	POD 1 ∼ POD3 (3 days)	Press 1–2 min/point QID, alt ears next day, replace seeds if pain, moist… bilat ears	①⑦⑨	NA		
Si 2017	Ta‐VNS	Sham Ta‐VNS	Shenmen (TF4)	POD 8 h, 16 h, 36 h, 56 h	1.5 Hz, rectangular wave (RW), 30 min/session	①②⑨/QMS	POPC	1	10
Chen 2015	AIDN + BA	Sham AIDN	Shenmen (TF4), Knee (AH4), Sympathetic (AH6a), Subcortex (AT4)	POD 3 ∼ POD5 (3 days)	Continuous stimulation	①②⑨	POPC	7	31
							AAR	1	3
Tian 2015	AP		Shenmen (TF4), Sympathetic (AH6a), Subcortex (AT4)	POD‐1 ∼ POD2 (4 days)	Press 3–5 min/point, bilat, Preop: q2h, Postop: q30 min	①⑨⑩/RSS	POPC	7	16
Wei 2015	AP		Subcortex (AT4), Shenmen (TF4), Endocrine (CO18), Knee (AH4)	POD 0 ∼ POD5 (6 days)	Press 35 s/point, bilat, TID, alt ears next day	⑧/Sleep Quality	NA		
Zhang 2014	AP		Knee (AH4), Subcortex (AT4), Shenmen (TF4), Endocrine (CO18), Liver (CO12)	POD‐3 ∼ POD 3 (7 days)	Press 3–5 min/point, bilat, TID, alt ears next day	①②⑨	POPC	6	27
He 2013	AP		Knee Joint (AH4), Shenmen (TF4), Subcortex (AT4), Sympathetic (AH6a)	POD 0 ∼ POD 7 (8 days)	Press 3 min/point, surgical side only, QID	①②④⑨	POPC	13	55
Tian 2013	AP		Subcortex (AT4), Shenmen (TF4), Adrenal (TG2p), Hip (AH3), Knee (AH4), Ankle (AH2)	POD 0 ∼ POD 21 (22 days)	Press 0.5–1 min/point, TID‐QID; replace seeds q1wk alt ears	⑤/WOMAC/FIM	NA		
Chang 2012	AP	Sham AP	Shenmen (TF4), Subcortex (AT4)	POD 1 ∼ POD 3 (3 days)	Press 3 min/point, TID	①②/McGill	NA		
Tong 2010	AP		Subcortex (AT4), Shenmen (TF4), Sympathetic (AH6a)	POD‐1 ∼ POD 7 (8 days)	Press 3–5 min/point, Preop: q2h, Postop: q30 min	①②④⑤⑨	POPC	4	11

*Note:* Serial numbers in outcome: ① Visual Analog Scale for Pain (VAS); ② Additional Painkillers; ③ Pressure Pain Threshold; ④ Hospital for Special Surgery Score (HSS); ⑤ Range of Motion (ROM); ⑥ Limb Swelling; ⑦ Serum Tests; ⑧ Numerical Rating Scale for Pain (NRS); ⑨ Adverse Reactions; ⑩ Satisfaction. AIDN: auricular intradermal needling. Mox: Moxibustion. PDV: Postoperative Drainage Volume Hemorheology. McGill: McGill Pain Questionnaire. WOMAC: Western Ontario and McMaster Universities Osteoarthritis Index. AAR: Adverse Auricular Acupoint Therapy Reaction. POPC: Postoperative Complications.

Abbreviations: AP, auricular acupressure; BA, body acupuncture; CMTT, Chinese medicine transdermal therapy; DDP, duration of drain placement; DVT, deep vein thrombosis; FIM, functional independence measure; Hb, hemoglobin level; PLOS, postoperative length of stay; QMS, quadriceps muscle strength; RSS, Ramsay Sedation Scale; Ta‐VNS, transcutaneous auricular vagus nerve stimulation; TFA, time to first ambulation; TFBM, time to first bowel movement; TFF, time to first flatus.

### 4.3. Risk of Bias Assessment

**FIGURE 3:**
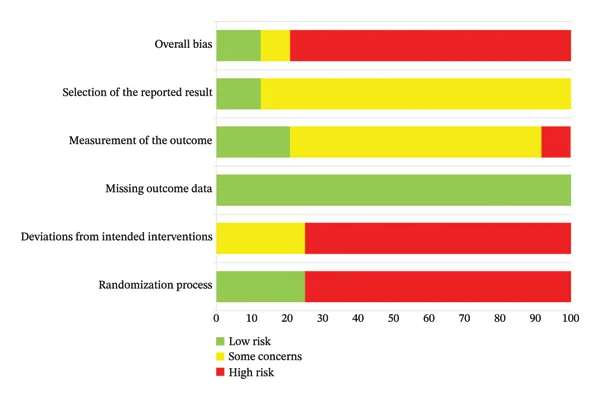
Overall risk of bias summary.

The risk of bias assessment showed that 20 studies were rated as “high risk,” 2 studies had “some concerns,” and 2 studies were judged as “low risk” for overall bias (Figure 3). A more detailed breakdown of the bias assessment for each study is provided in Supporting Information 1: Figure S1.

### 4.4. Meta‐Analysis of the Results

#### 4.4.1. AART vs. RRT: POD1 VAS‐R

A total of 11 studies [12, 20–22, 25, 27, 30, 32, 33, 36] involving 844 patients were pooled using a random‐effects model (I^2^ = 76%). The meta‐analysis showed that AART as an adjunct to RRT significantly reduced resting pain scores on postoperative day 1 (POD1 VAS‐R) compared with RRT alone [mean difference (MD) = −0.38, 95% confidence interval (CI) (−0.58, −0.18), *p* < 0.01, low certainty]. Although the funnel plot appeared generally symmetrical, Egger’s test suggested potential publication bias (*t* = −2.62, df = 9, *p* = 0.028); this apparent discrepancy between the visual inspection and statistical test may reflect the limited power of funnel plot asymmetry tests with small numbers of studies, or the presence of small‐study effects that are not readily apparent from visual inspection alone. Given the statistical evidence of potential publication bias, the effect estimate for POD1 VAS‐R may be overestimated, and these results should be interpreted with particular caution. Sensitivity analysis confirmed the robustness of the results (effect size range: −0.43 to −0.34). Subgroup analysis indicated that AP had significant effects [MD = −0.55, 95% CI (−0.94, −0.16), *p* < 0.01, very low certainty], whereas AIDN and Ta‐VNS showed non‐significant effects (*p* > 0.05, low certainty). CT also demonstrated significant effectiveness [MD = −0.23, 95% CI (−0.44, −0.03), *p* < 0.05, very low certainty], with significant differences between subgroups (*p* < 0.01). Detailed analysis results and tree diagrams are presented in Figure 4A, and the GRADE evidence quality assessment is available in Supporting Information 2.

FIGURE 4Forest Plots for AART vs. RRT/Sham on VAS after TKA. Note: (A) AART vs. RRT for POD1 VAS‐R. (B) AART vs. RRT for POD3 VAS‐R. (C) AART vs. RRT for POD7 VAS‐R. (D) AART vs. Sham for POD1 VAS‐R. (E) AART vs. Sham for POD3 VAS‐R. (F) AART vs. RRT for POD1 VAS‐M. (G) AART vs. RRT for POD3 VAS‐M. (H) AART vs. RRT for POD7 VAS‐M.

**Figure:**
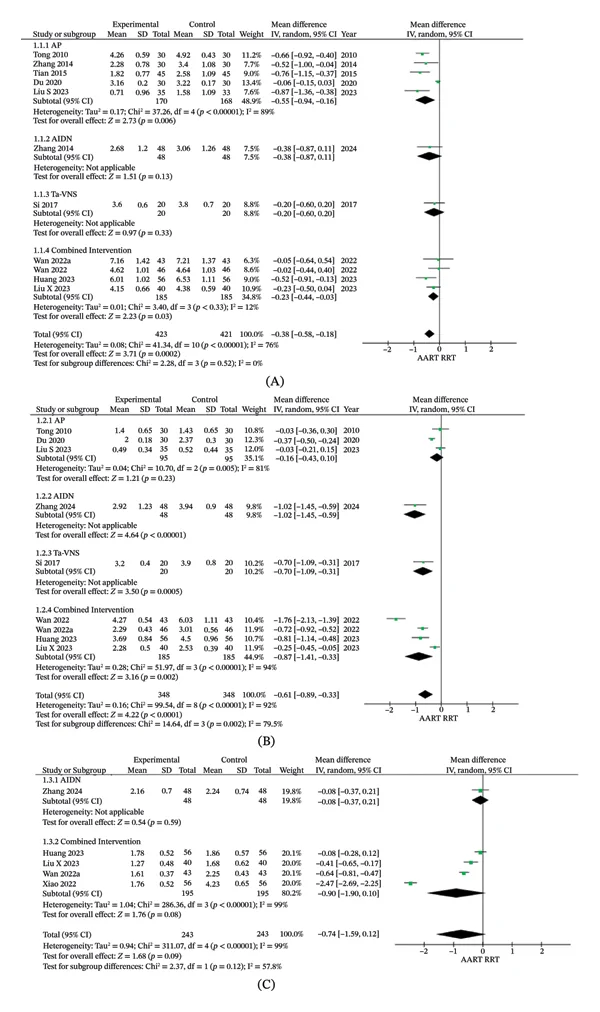

**Figure:**
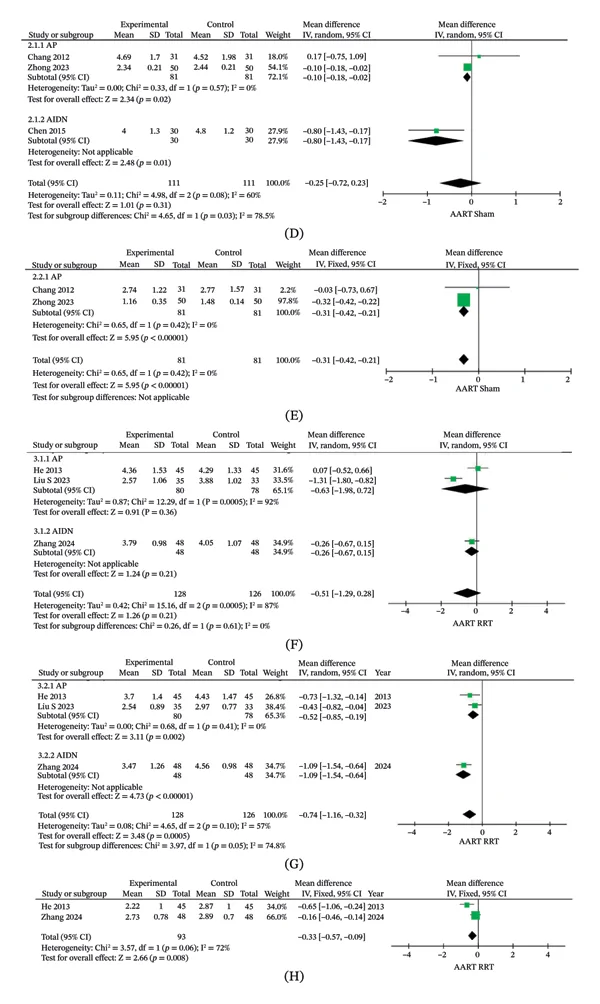

#### 4.4.2. AART vs. RRT: POD3 VAS‐R

A pooled analysis of 9 studies [12, 21, 25, 27, 30, 32, 33, 36] involving 696 patients, using a random‐effects model (I^2^ = 92%), showed that AART significantly reduced resting pain scores on postoperative day 3 (POD3 VAS‐R) compared with RRT [MD = −0.61, 95% CI (−0.89, −0.33), *p* < 0.01, low certainty]. The funnel plot was generally symmetrical (Figure 5B), and sensitivity analysis confirmed the robustness of the results (effect size range: −0.69 to −0.47). To address the extremely high heterogeneity in the POD3 VAS‐R outcome, we re‐examined it using a leave‐one‐out sensitivity analysis. The results showed that after excluding Wan 2022, I^2^ decreased from 92% to 85%, and the MD changed from −0.61 to −0.46. However, excluding any single study failed to reduce I^2^ below 80%. This suggests that the heterogeneity primarily stems from systematic differences across studies rather than being driven by any individual outlier study. Subgroup analysis revealed significant differences between intervention types (*p* < 0.01). AP showed no significant effect (*p* > 0.05, very low certainty), whereas AIDN significantly reduced pain (*p* < 0.01, moderate certainty). Ta‐VNS also demonstrated significant pain reduction (*p* < 0.01, very low certainty), and CT showed significant efficacy [MD = −0.87, 95% CI (−1.41, −0.33), *p* < 0.01, very low certainty]. Detailed results are presented in Figure 4B, and the GRADE evidence profile is available in Supporting Information 2.

FIGURE 5Funnel plots for publication bias (AART vs. RRT/Sham on VAS). Note: (A) AART vs. RRT for POD1 VAS‐R. (B) AART vs. RRT for POD3 VAS‐R. (C) AART vs. RRT for POD7 VAS‐R. (D) AART vs. Sham for POD1 VAS‐R. (E) AART vs. Sham for POD3 VAS‐R. (F) AART vs. RRT for POD1 VAS‐M. (G) AART vs. RRT for POD3 VAS‐M. (H) AART vs. RRT for POD7 VAS‐M.

**Figure:**
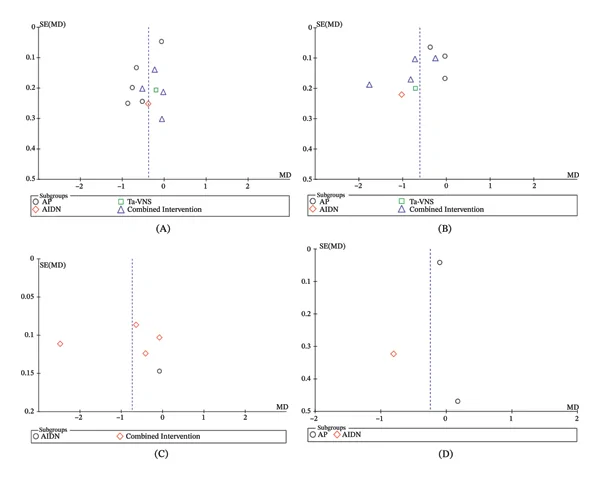

**Figure:**
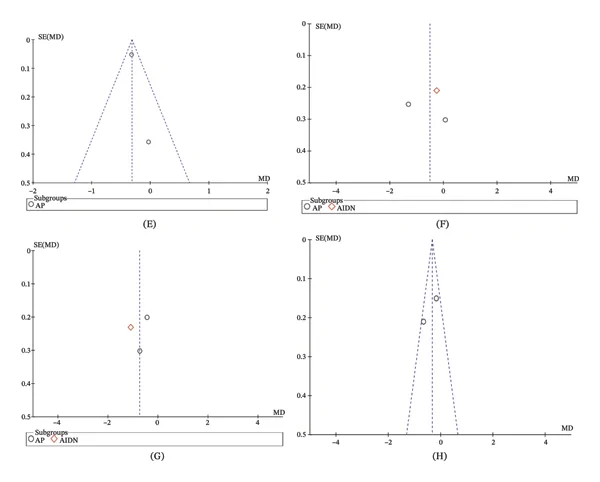

#### 4.4.3. AART vs. RRT: POD7 VAS‐R

A pooled analysis of 5 studies [12, 26, 27, 29, 33] involving 486 patients was conducted using a random‐effects model (I^2^ = 99%). The results showed no statistically significant difference in resting pain scores on postoperative day 7 (POD7 VAS‐R) between the AART and RRT groups [MD = −0.74, 95% CI (−1.59, 0.12), *p* > 0.01, very low certainty]. For the POD7 VAS‐R outcome, which exhibited extremely high heterogeneity, the leave‐one‐out sensitivity analysis showed that after excluding Xiao 2022, I^2^ decreased from 99% to 86%, and the MD changed from −0.74 to −0.31. However, excluding any single study failed to reduce I^2^ below 80%, indicating that the heterogeneity is primarily attributable to systematic differences across studies rather than being driven by any individual outlier study. Due to the limited number of studies, the funnel plot was used only to visualize the distribution of studies (Figure 5C), and no formal assessment of publication bias was performed. Sensitivity analysis indicated that excluding the Xiao 2022 study resulted in a statistically significant effect size of −0.31, whereas the exclusion of other individual studies did not change the results. Heterogeneity remained extremely high throughout sensitivity analyses (I^2^ > 84%), suggesting that the findings are heavily influenced by individual studies and that the conclusions have low reliability. Subgroup analysis results are presented in Figure 4C, with detailed GRADE assessments available in Supporting Information 2.

#### 4.4.4. AART vs. Sham: VAS‐R

Three studies [31, 38, 40] compared the effects of AART with sham auricular therapy on VAS‐R after TKA. The results showed no statistically significant difference between the two groups on POD1 (*p* > 0.05, very low certainty). However, AART significantly reduced VAS‐R on POD3 [MD = −0.31, 95% CI (−0.42, −0.21), *p* < 0.05, moderate certainty]. Because of the limited number of studies, funnel plots were used only to visually represent the distribution of studies rather than for formal assessment of publication bias (Figure 5D, E). Subgroup analysis results and tree diagrams are presented in Figure 4D, E, with detailed GRADE assessments available in Supporting Information 2.

#### 4.4.5. AART vs. RRT: VAS‐M

Three studies [12, 32, 39] involving 254 patients evaluated the effects of AART versus RRT on movement‐evoked visual analog scale (VAS‐M) scores at different time points following TKA. The results showed no significant difference in VAS‐M scores between the groups on postoperative day 1 (POD1) (*p* > 0.05, very low certainty). However, AART significantly reduced VAS‐M scores on POD3 [MD = −0.74, 95% CI (−1.16, −0.32), *p* < 0.01, moderate certainty] and POD7 [MD = −0.33, 95% CI (−0.57, −0.09), *p* < 0.01, moderate certainty]. Because of the limited number of studies, funnel plots were used only to visualize the distribution of studies rather than for formal assessment of publication bias (Figure 5F–H). Subgroup analysis results and tree diagrams are presented in Figure 4F–H, with detailed GRADE assessments available in Supporting Information 2.

#### 4.4.6. AART vs. RRT/Sham: Hospital for Special Surgery (HSS)

Eight studies [21, 26, 27, 30, 31, 33, 39] analyzed the effects of AART versus RRT or sham on HSS scores at different time points following TKA. The results showed no significant differences in HSS scores between the groups on POD1 (very low certainty) and POD14 (low certainty) (*p* > 0.05). However, AART significantly improved HSS scores on POD3 (very low certainty), POD7 (low certainty), POD30 (very low certainty), and POD90 (low certainty). Because of the limited number of studies, funnel plots were used only to visualize the distribution of studies rather than for formal assessment of publication bias (Supporting Information‐1: Figure S2). Detailed analysis results and tree diagrams are presented in Supporting Information 1: Figure S3, with comprehensive GRADE assessments available in Supporting Information 2.

#### 4.4.7. AART vs. RRT: Painkiller

Eight studies [13, 21, 22, 32, 36, 38–40] investigated the impact of AART versus RRT on postoperative painkiller consumption in TKA patients. The results showed no significant differences between groups in the use of Step 3 analgesics (low certainty) or patient‐controlled analgesia (PCA) opioid consumption on postoperative day 2 (very low certainty) (*p* > 0.05). However, AART significantly reduced the consumption of Step 2 analgesics (*p* < 0.01, very low certainty). Because of the limited number of studies, funnel plots were used only to visualize the distribution of studies rather than for formal assessment of publication bias (Supporting Information‐1: Figure S4). Detailed analysis results and tree diagrams are presented in Supporting Information 1: Figure S5, with comprehensive GRADE assessments available in Supporting Information 2.

#### 4.4.8. AART vs. RRT: AROM

Eight studies [21, 24, 30, 32–34, 39] evaluated the effect of AART versus RRT on active range of motion (AROM) following TKA. The results showed that AART significantly improved AROM on postoperative day 7 (very low certainty) and day 14 (very low certainty) (*p* < 0.01). Because of the limited number of studies, funnel plots were used only to visualize the distribution of studies rather than for formal assessment of publication bias (Supporting Information‐1: Figure S4). Detailed analysis results and tree diagrams are presented in Supporting Information 1: Figure S5, with comprehensive GRADE assessments available in Supporting Information 2.

#### 4.4.9. AART vs. RRT: PRAEs

We also analyzed postoperative complications (PRAEs) across the relevant studies. Eight studies [20–22, 25, 33, 36, 38, 39] showed that AART significantly reduced the incidence of postoperative vomiting (PV) (*p* < 0.01, moderate certainty). Publication bias was assessed using a funnel plot, which appeared visually symmetrical (Supporting Information 1: Figure S6A). Leave‐one‐out sensitivity analysis indicated highly stable results; regardless of which study was omitted, the pooled risk ratio (RR) remained between 0.27 and 0.30, and all 95% CIs were below 1, indicating statistical significance. Heterogeneity was consistently absent (I^2^ = 0%). Other analyses showed no significant differences between groups in the incidence of postoperative pruritus or hematoma (*p* ≥ 0.05, very low certainty). However, AART significantly reduced the incidence of deep vein thrombosis (DVT) (*p* < 0.05, low certainty), postoperative dizziness (*p* < 0.01, low certainty), and urinary retention (*p* < 0.01, moderate certainty). Because of the limited number of studies reporting these PRAEs, funnel plots were used only to visualize the distribution of studies rather than for formal assessment of publication bias (Supporting Information‐1: Figure S6B-F). Detailed results and tree diagrams for all PRAEs are presented in Supporting Information 1: Figure S7, with GRADE certainty assessments available in Supporting Information 2.

### 4.5. Supplemental Sensitivity Analysis

To assess the robustness of our findings to the predominance of high‐risk studies, we performed sensitivity analyses by excluding all 20 studies rated as high risk of bias and re‐analyzing outcomes that had at least two non‐high‐risk studies available for pooling. Among the five non‐high‐risk studies (3 low risk, 2 some concerns), only VAS pain scores and certain analgesic consumption outcomes had sufficient data for quantitative synthesis; all other outcomes, including AROM and HSS scores, had fewer than two high‐quality studies and could not be meaningfully pooled. The sensitivity analyses showed:

POD1 VAS‐R (2 studies): MD = −0.41, 95% CI (−0.76, −0.06), *p* = 0.02, consistent with the main analysis in direction;

POD3 VAS‐R (3 studies): MD = −0.56, 95% CI (−1.53, 0.40), *p* = 0.25, directionally consistent but crossing the null;

POD1 VAS‐M (2 studies): MD = −0.15, 95% CI (−0.49, 0.18), *p* = 0.37, consistent with the main analysis;

POD3 VAS‐M (2 studies): MD = −0.96, 95% CI (−1.32, −0.60), *p* < 0.001, consistent with the main analysis;

POD7 VAS‐M (2 studies): MD = −0.38, 95% CI (−0.86, 0.10), *p* = 0.12, consistent with the main analysis;

Step 3 opioids (2 studies): MD = −7.16, 95% CI (−16.49, 2.17), *p* = 0.13, directionally consistent but crossing the null.

For all other outcomes, the number of non‐high‐risk studies was insufficient (fewer than 2) to permit meaningful sensitivity analyses, and the potential influence of high‐risk studies on these results could not be excluded.

### 4.6. Network Meta‐Analysis

**FIGURE 6:**
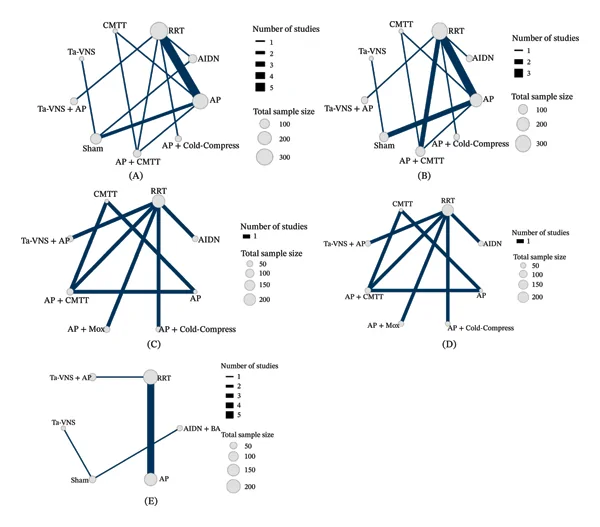
Network graphs of comparisons for different outcomes. Note: (A) Network for POD1 VAS‐R. (B) Network for POD3 VAS‐R. (C) Network for POD7 VAS‐R. (D) Network for POD7 AROM. (E) Network for postoperative vomiting.

Among the 24 studies included in the analysis, 14 studies [12, 20–22, 25, 27, 29–33, 36, 38, 40] (9 interventions) used POD1 VAS‐R as an outcome measure; 12 studies [12, 21, 25, 27–33, 36, 40] (9 interventions) used POD3 VAS‐R; 6 studies [12, 26–29, 33] (9 interventions) used POD7 VAS‐R; 5 studies [24, 28, 29, 32, 33] (5 interventions) used POD7 AROM; and 8 studies [20–22, 25, 33, 36, 38, 39] (5 interventions) reported the incidence of PV following TKA. The network relationships for each outcome are shown in Figure 6. Notably, the network for PV was disconnected (Figure 6E) and therefore was not included in the NMA.

#### 4.6.1. POD1 VAS‐R

Fourteen studies were included in the NMA for POD1 VAS‐R. AP combined with Chinese medicine transdermal therapy (AP + CMTT) was ranked as the most potentially effective intervention, achieving the highest SUCRA value of 0.90 (Supporting Information 1: Figure S8A). Compared with RRT, this combination therapy significantly reduced pain scores, with a MD of −1.87 (95% CrI: −3.62, −0.13). The model consistency assessment indicated no significant inconsistency (ΔDIC = 3.5). Local inconsistency, evaluated using the node‐splitting method across six key comparisons, revealed no significant inconsistencies (all Bayesian *p*‐values > 0.05). For the Bayesian NMA, a random‐effects model was fitted with 4 MCMC chains, each comprising 20,000 iterations with a burn‐in of 5000 iterations. Convergence was assessed using the Brooks–Gelman–Rubin diagnostic (potential scale reduction factor < 1.05 for all parameters). The DIC was used for model comparison.

#### 4.6.2. POD3 VAS‐R

Twelve studies were included in the NMA for POD3 VAS‐R outcomes. AP + CMTT was ranked as the most potentially effective intervention (SUCRA = 0.73, Supporting Information 1: Figure S8B). However, its comparison with RRT showed a non‐significant effect, with an MD of −0.83 (95% CrI: −1.78, 0.07). Despite the lack of statistical significance, the point estimate suggests a beneficial trend. The model showed low global inconsistency (ΔDIC = 1.7), and local node‐splitting analysis revealed no significant inconsistencies (all Bayesian *p*‐values > 0.05).

#### 4.6.3. POD7 VAS‐R

Six studies were included in the NMA for POD7 VAS‐R outcomes. AP combined with moxibustion (AP + Mox) ranked highest in analgesic efficacy (SUCRA = 0.83, Supporting Information 1: Figure S8C). However, its comparison with RRT did not reach statistical significance, with an MD of −2.28 (95% CrI: −8.07, 3.98). Despite this, the point estimate for AP + Mox was more favorable than those of all other interventions. Model consistency assessment indicated no significant inconsistency (ΔDIC = 0.4). Due to the simplicity of the network structure, which included only six studies, no closed loops were available for node‐splitting analysis.

#### 4.6.4. POD7 AROM

Five studies were included in the NMA for POD7 AROM outcomes. AP + CMTT was ranked as the most potentially effective intervention (SUCRA = 0.83, Supporting Information 1: Figure S8D). However, its comparison with RRT did not show a statistically significant effect, with an MD of 8.29 (95% CrI: −4.00, 20.44). No significant inconsistency was detected in the model (ΔDIC = 0.7), and local node‐splitting analysis revealed no significant inconsistencies (all Bayesian *p*‐values > 0.05).

It is important to note that the statistical significance of NMA results may differ from pairwise meta‐analyses for the same outcome, primarily due to reduced statistical power when data are stratified into multiple specific intervention subgroups. For example, while pairwise meta‐analysis showed that AART significantly reduced POD3 VAS‐R compared with RRT (MD = −0.61, *p* < 0.01), the NMA comparison of the top‐ranked intervention (AP + CMTT) versus RRT did not reach statistical significance (MD = −0.83, 95% CrI: −1.78, 0.07). This discrepancy likely reflects the reduced sample size at individual nodes in the NMA (only 2 studies contributed to the AP + CMTT vs. RRT comparison for POD3 VAS‐R), rather than a true contradiction between the two analytical approaches. Therefore, SUCRA rankings should be interpreted primarily as exploratory and hypothesis‐generating, and the wide CrIs around NMA estimates should be carefully considered when interpreting the relative rankings.

## 5. Discussion

This study evaluated the effects of AART on rehabilitation outcomes following TKA. The main results showed that, for pain control, AART significantly reduced resting pain scores on postoperative days 1 and 3 (POD1 and POD3 VAS‐R) compared with RRT alone, whereas no significant difference was observed on day 7. For movement‐evoked pain (VAS‐M), AART led to significant improvements on POD3 and POD7. Additionally, AART reduced the consumption of certain analgesics. Regarding functional recovery, AART improved HSS scores on POD3, 7, 30, and 90 and enhanced AROM on POD7 and 14. In terms of complications, AART significantly reduced the incidence of PV, DVT, dizziness, and urinary retention. Subgroup analyses suggested that AP and CT may be more effective for specific outcomes. Network meta‐analysis further indicated that AP combined with CMTT (AP + CMTT) could represent the optimal intervention for early resting pain. However, the certainty of evidence for most outcomes was low or very low. Some analyses were potentially influenced by publication bias or substantial heterogeneity, highlighting the need for cautious interpretation of these findings.

### 5.1. Pain Management

Postoperative pain significantly impedes the recovery of knee function and quality of life in patients following TKA, while also imposing a substantial economic burden [41]. This study demonstrates that AART provides consistent improvements in early postoperative resting pain (POD1 and POD3) and movement‐evoked pain, while also reducing the requirement for certain analgesics, supporting its use as an adjunctive analgesic strategy after surgery. Notably, the high‐frequency auricular points identified in our analysis (Shenmen, Subcortex, Knee, Sympathetic; see Figure 2) are located in regions of the auricle with the densest and most complex neural distribution, primarily innervated by mixed branches of the vagus, glossopharyngeal, and facial nerves [42]. Recent research suggests that Ta‐VNS may represent a key mechanism through which AART exerts its analgesic effects [43]. AART may activate critical nuclei in the endogenous descending pain modulatory system such as the ventral tegmental area, nucleus tractus solitarius, and raphe nuclei by stimulating the vagus nerve, thereby inhibiting nociceptive signal transmission and reducing pain perception [44]. Additionally, animal studies indicate that vagus nerve stimulation can modulate neuroinflammation in the hippocampus, subsequently altering pain sensitivity [45]. However, the nonsignificant analgesic effect of AART observed on postoperative day 7 suggests that short‐term auricular stimulation may be insufficient to maintain long‐term analgesia. Previous research has shown that acupoint interventions can induce neuroplasticity [46]. Therefore, we hypothesize that prolonged and consistent AART may lead to remodeling of neural circuits, influencing the transmission and processing of pain signals. Future studies should investigate whether extended AART intervention durations can help alleviate chronic postoperative pain.

Furthermore, the clinical significance of the observed pain reduction warrants careful consideration. The pooled effect sizes for VAS‐R reduction—MD of −0.38 on POD1 and −0.61 on POD3—were modest in magnitude but should be interpreted from the following perspectives. In terms of effect additivity, as an adjunct to multimodal analgesia, AART provides additional benefit beyond conventional analgesia (including opioids, PCA, etc.). This incremental reduction of 0.38–0.61 cm may translate into indirect clinical value by reducing the need for rescue analgesia. In terms of comprehensive benefits, beyond the direct effect on pain scores, AART also reduced the incidence of PV (RR = 0.28), dizziness (RR = 0.28), and urinary retention (RR = 0.33). The reduction in these complications may indirectly improve patients’ overall recovery experience, and the combined clinical significance may extend beyond the reduction in pain scores alone. In terms of cumulative effects, although the effect size at each individual time point was modest, the sustained improvement in pain scores (significant at both POD1 and POD3) may facilitate early functional exercise through cumulative effects, thereby exerting a potential impact on long‐term functional recovery.

### 5.2. Functional Recovery

The recovery of joint function following TKA is a critical determinant of patients’ quality of life. This study confirms that the AART rehabilitation protocol significantly improves HSS scores on postoperative days 3, 7, 30, and 90, enhances AROM on days 7 and 14, and substantially reduces the incidence of surgery‐ or anesthesia‐related complications. It is important to note that joint function recovery depends not only on postoperative pain management but also on multiple factors, including swelling control, muscle strength, psychological status, and complication prevention [47–49]. AART likely acts through integrated multi‐target and multi‐pathway mechanisms. Beyond its analgesic effects, it may further contribute to functional recovery by reducing local swelling, enhancing muscle strength, improving patients’ rehabilitation confidence, and lowering complication risks. This perspective is supported by several clinical studies demonstrating benefits such as alleviating perioperative anxiety‐induced insomnia [50], promoting resolution of surgical site swelling [51], and improving postoperative gastrointestinal dysfunction [52, 53]. However, there remains a lack of rigorously designed, large‐sample RCTs to validate the overall efficacy of AART in functional recovery after TKA and to elucidate its specific mechanisms. Future high‐quality research is needed to clarify its pathways of action and provide evidence‐based support for integrating AART into enhanced recovery after surgery (ERAS) protocols in orthopedics.

### 5.3. Exploration of the Optimal Solution

For early postoperative resting pain, this NMA identified AP combined with directional CMTT (AP + CMTT) as the potentially optimal intervention. Although this combination demonstrated the highest SUCRA ranking, suggesting possible synergistic effects supported by multiple direct comparison studies [29, 31] and aligning with the traditional Chinese medicine theoretical framework of “combined acupuncture and herbal medicine, treating both internally and externally,” the evidence remains insufficient to establish it as a definitive optimal regimen. The lack of standardization in the composition of drug patches leads to uncertainty regarding their specific therapeutic effects; additionally, variations in the selection of transdermal administration sites, inconsistencies in methods to promote drug penetration, and potential biases in the original studies further undermine the reliability of the relevant evidence. Therefore, the primary contribution of this finding lies in proposing a promising “preferred hypothesis,” which warrants future validation through rigorously designed RCTs. At the same time, the SUCRA rankings from our NMA should be interpreted with caution for several reasons: first, the wide CrIs around many NMA estimates indicate substantial uncertainty in the relative rankings; second, the rankings are sensitive to the specific network structure and the studies included, and the addition of new studies could substantially alter the hierarchy; and third, the highest‐ranked intervention (AP + CMTT) was supported by a limited number of direct comparisons, limiting the stability of its ranking. In summary, the primary value of these rankings is to generate hypotheses for future head‐to‐head comparative trials rather than to provide definitive clinical recommendations.

### 5.4. Limitations

This study has several limitations. First, the quality of evidence for most outcomes was rated as “low” or “very low,” primarily due to the prevalent risk of bias in the included studies—20 of 24 studies were rated as high risk of bias—combined with substantial heterogeneity across outcome measures and imprecision in effect estimates. Consequently, we have moderate‐to‐low confidence that the pooled effect estimates reflect true treatment effects rather than systematic biases. The consistent direction of effects across multiple outcomes and time points provides some reassurance, but the possibility of overestimation due to methodological limitations cannot be excluded.

When interpreting the core findings, the considerable heterogeneity warrants particular attention to its potential clinical sources. Specifically, differences in postoperative baseline analgesic protocols represent a key confounding factor. Although the control groups in all included studies were defined as “routine rehabilitation treatment” (RRT), the specific content of “routine” varied considerably across research centers. For example, routine protocols in some centers may include standardized PCA pumps and multimodal oral analgesics, whereas other centers may rely solely on on‐demand analgesic administration. This lack of standardization in control interventions directly affects the comparability of effect sizes between AART and RRT, thereby introducing clinical heterogeneity.

Second, the diversity of rehabilitation pathways cannot be overlooked. Variations in the initiation timing of physical therapy, daily frequency, specific content (e.g., passive versus active exercises), and the application of adjunctive measures such as cryotherapy may all influence functional recovery outcomes, including AROM and HSS scores. Third, subtle differences in the timing of outcome assessments constitute an important source of heterogeneity. Although we performed time‐point subgroup analyses based on postoperative days (e.g., POD1, POD3), the specific assessment timing down to the hour may vary across studies. For example, POD1 assessments may be performed exactly 24 h post‐surgery or during morning rounds on the first postoperative day. For indicators such as pain, which fluctuate considerably during the acute phase, differences of just a few hours can lead to significantly different scores.

Furthermore, details of the AART interventions themselves—such as acupoint selection, stimulation frequency, duration of single pressure applications, and total intervention cycles—were not fully standardized across the original studies. The diversity of AART interventions included in this review, ranging from AP to transcutaneous vagus nerve stimulation to various combination therapies, represents a double‐edged sword: on one hand, it reflects the breadth of clinical applications of AART in TKA rehabilitation; on the other hand, it introduces substantial clinical heterogeneity that complicates pooled interpretation. Importantly, even within the same intervention category (e.g., AP), there was considerable variation in acupoint selection, stimulation frequency, duration of application, and frequency of seed replacement. This lack of standardization likely contributed to the substantial heterogeneity observed in several outcomes. Although we explored these factors through subgroup and NMA, they remain potential sources of heterogeneity.

Several analyses were based on a limited number of studies (e.g., POD7 VAS‐R: 5 studies; POD1/3 VAS‐M: 3 studies each), which compromises statistical power and the stability of effect estimates. Additionally, significant publication bias was detected for POD1 VAS‐R (Egger’s test, *p* = 0.028), suggesting that unpublished negative results may exist, potentially leading to overestimation of the pooled effect size. Although trim‐and‐fill adjustment was not performed due to the small number of studies, the potential for overestimation should be considered when interpreting the primary findings. For outcomes with fewer than 10 studies, formal publication bias tests were not performed, but the possibility of selective reporting remains. Certain other analyses (e.g., comparisons with sham points) and specific nodes in the NMA were also based on a limited number of studies, undermining the stability of the corresponding results.

### 5.5. Recommendations for Future Research

Future primary research should aim to improve the standardization and transparency of intervention protocols and control measures, as well as unify assessment procedures for key outcome indicators, specifying operational details such as acupoint selection and stimulation parameters, in order to reduce clinical heterogeneity and draw more reliable conclusions. Meanwhile, registration of clinical trials and publication of all results regardless of direction or magnitude of effects should be encouraged to mitigate publication bias. Future studies should prioritize large‐sample, multicenter, and methodologically rigorous RCTs, with particular emphasis on comparative effectiveness between combination therapies and other interventions. In addition, systematic evaluation of long‐term functional outcomes, patient‐reported outcomes, and health economic benefits should be strengthened, and the mechanisms underlying AART warrant in‐depth investigation to provide a robust scientific foundation for its broader clinical application.

## 6. Conclusion

Based on current evidence, AART, particularly in combined intervention forms, can serve as an effective non‐pharmacological adjunct within multimodal rehabilitation protocols following TKA. It shows potential in alleviating acute pain, promoting functional recovery, and reducing related complications. In clinical practice, incorporating AART modalities, such as AP, into standard postoperative care pathways for TKA is recommended where feasible, thereby diversifying rehabilitation strategies. However, the overall quality of current evidence is low, and the above conclusions should be interpreted with caution, serving only as preliminary clinical reference, pending further validation by high‐quality future research.

**Nomenclature**
AART: Auricular acupoint‐related therapy
TKA: Total knee arthroplasty
RCT: Randomized controlled trial
AP: Auricular acupressure
AIDN: Auricular intradermal needling
Ta‐VNS: Transcutaneous auricular vagus nerve stimulation
AA: Auricular acupuncture
CMTT: Chinese medicine transdermal therapy
CT: Combination therapy
RRT: Routine rehabilitation treatment
POD num: Postoperative day number
VAS‐R: Visual analog scale for resting pain
VAS‐M: Movement‐evoked visual analog scale
HSS: Hospital for special surgery knee score
AROM: Active range of motion
PRAE: Postoperative complication
PV: Postoperative vomiting
TCM: Traditional Chinese Medicine

## Author Contributions

Qindong Mi, Yunxi Xu: conceptualization, methodology, formal analysis, investigation, visualization, writing–original draft, data curation; Fei Bao: conceptualization, methodology, project administration, supervision, resources, writing–review and editing, and funding acquisition; Xiaolong Zhao: conceptualization, methodology, investigation, and validation; Lijin Liu: conceptualization, methodology, and software; Zhihong Wu: conceptualization, methodology, and funding acquisition.

## Funding

This work was supported by (1) Sedimentation Research Funding Integration Entrustment Project, Peking Union Medical College Hospital (No. ZC201903189) and (2) National High‐Level Hospital Clinical Research Funding (No. 2025‐PUMCH‐C‐002).

## Ethics Statement

The authors have nothing to report.

## Conflicts of Interest

The authors declare no conflicts of interest.

## Supporting Information

Additional supporting information can be found online in the Supporting Information section.

## Supporting information


**Supporting Information 1** This file contains the following: search strategy; overview of complementary therapy interventions; risk of bias assessment (Figure S1); forest plot and funnel plot for supporting analyses (Figure S2–S7); and SUCRA curves (Figure S8).

**Supporting Information 2** This file contains the following: GRADE evidence profiles for all outcome measures.

**Supporting Information 3** PRISMA 2020 checklist714.

## Data Availability

All data supporting the findings of this systematic review and meta‐analysis were extracted from the published studies included in our article. These source articles are listed in the Reference section and are publicly available through their respective publishers.
