# Supplementary material for: Does Auricular Acupoint‐Related Therapy Enhance Rehabilitation After Total Knee Arthroplasty? A Systematic Review and Network Meta‐Analysis
Source: Pain Res Manag. 2026 Sep 20;2026:9277186. doi: 10.1155/prm/9277186 (PMC13591118; doi:10.1155/prm/9277186)
Supplement: Supplementary file 1 [file PRM-2026-9277186-s002.pdf]

Supplementary materials-2：

Summary of Findings and Certainty of Evidence Assessments

Bibliography: AART vs. RRT in TKA Rehab: VAS-R Comparison

Quality assessment							Summary of Findings				
**Participants (studies) Follow up**	**Risk of bias**	**Inconsistency**	**Indirectness**	**Imprecision**	**Publication bias**	**Overall quality of evidence**	**Study event rates (%)**		**Relative effect** (95% CI)	**Anticipated absolute effects**	
							**With RRT on VAS-R scale**	**With AART**		**Risk with RRT on VAS-R scale**	**Risk difference with AART** (95% CI)
**1VAS-R** (IMPORTANT OUTCOME; Better indicated by lower values)											
844 (11 studies)	no serious risk of bias	no serious inconsistency	very serious[1]	no serious imprecision[2]	undetected	⊕⊕⊖⊖ **LOW**[1,2] due to indirectness	421	423	-		The mean 1vas-r in the intervention groups was **0.38 lower** (0.58 to 0.18 lower)
**1VAS-R - AP** (IMPORTANT OUTCOME; Better indicated by lower values)											
338 (5 studies)	no serious risk of bias	serious[3]	very serious	no serious imprecision[2]	undetected	⊕⊖⊖⊖ **VERY LOW**[2,3] due to inconsistency, indirectness	168	170	-		The mean 1vas-r - ap in the intervention groups was **0.55 lower** (0.94 to 0.16 lower)
**1VAS-R - AIDN** (Better indicated by lower values)											
96 (1 study)	no serious risk of bias	very serious[4]	no serious indirectness	no serious imprecision	undetected	⊕⊕⊖⊖ **LOW**[4] due to inconsistency	48	48	-		The mean 1vas-r - aidn in the intervention groups was **0.38 lower** (0.87 lower to 0.11 higher)
**1VAS-R - Ta-VNS** (IMPORTANT OUTCOME; Better indicated by lower values)											
40 (1 study)	no serious risk of bias	very serious[3,4]	very serious[1]	no serious imprecision	undetected	⊕⊖⊖⊖ **VERY LOW**[1,3,4] due to inconsistency, indirectness	20	20	-		The mean 1vas-r - ta-vns in the intervention groups was **0.2 lower** (0.6 lower to 0.2 higher)
**1VAS-R - Combined Intervention** (Better indicated by lower values)											
370 (4 studies)	no serious risk of bias	very serious[3,4]	very serious[1]	no serious imprecision	undetected	⊕⊖⊖⊖ **VERY LOW**[1,3,4] due to inconsistency, indirectness	185	185	-		The mean 1vas-r - combined intervention in the intervention groups was **0.23 lower** (0.44 to 0.03 lower)
**3 VAS-R** (Better indicated by lower values)											
696 (9 studies)	no serious risk of bias	no serious inconsistency	very serious[1]	no serious imprecision[2]	undetected	⊕⊕⊖⊖ **LOW**[1,2] due to indirectness	348	348	-		The mean 3 vas-r in the intervention groups was **0.61 lower** (0.89 to 0. 33 lower)
**3 VAS-R - AP** (Better indicated by lower values)											
190 (3 studies)	no serious risk of	very serious[3,4]	very serious[1]	no serious imprecision[2]	undetected	⊕⊖⊖⊖ **VERY LOW**[1,2,3,4]	95	95	-		The mean 3 vas-r - ap in the

	bias					due to inconsistency, indirectness			intervention groups was **0.15 lower** (0.39 lower to 0.09 higher)
**3 VAS-R - AIDN** (Better indicated by lower values)									
96 (1 study)	no serious risk of bias	serious[3]	no serious indirectness	no serious imprecision	undetected	⊕⊕⊕⊖ **MODERATE**[3] due to inconsistency	48 48	-	The mean 3 vas-r - aidn in the intervention groups was **1.02 lower** (1.45 to 0.59 lower)
**3 VAS-R - Ta-VNS** (Better indicated by lower values)									
40 (1 study)	serious[5]	serious[3]	very serious[1]	no serious imprecision	undetected	⊕⊖⊖⊖ **VERY LOW**[1,3,5] due to risk of bias, inconsistency, indirectness	20 20	-	The mean 3 vas-r - ta-vns in the intervention groups was **0.7 lower** (1.09 to 0.31 lower)
**3 VAS-R - Combined Intervention** (Better indicated by lower values)									
370 (4 studies)	no serious risk of bias	serious[3]	very serious[1]	no serious imprecision[2]	undetected	⊕⊖⊖⊖ **VERY LOW**[1,2,3] due to inconsistency, indirectness	185 185	-	The mean 3 vas-r - combined intervention in the intervention groups was **0.87 lower** (1.41 to 0.33 lower)
**7 VAS-R** (Better indicated by lower values)									
486 (5 studies)	no serious risk of bias	serious[4]	very serious[1]	no serious imprecision[2]	undetected	⊕⊖⊖⊖ **VERY LOW**[1,2,4] due to inconsistency, indirectness	243 243	-	The mean 7 vas-r in the intervention groups was **0.74 lower** (1.59 lower to 0.12 higher)
**7 VAS-R - AIDN** (Better indicated by lower values)									
96 (1 study)	no serious risk of bias	very serious[3,4]	no serious indirectness	no serious imprecision	undetected	⊕⊕⊖⊖ **LOW**[3,4] due to inconsistency	48 48	-	The mean 7 vas-r - aidn in the intervention groups was **0.08 lower** (0.37 lower to 0.21 higher)
**7 VAS-R - Combined Intervention** (Better indicated by lower values)									
390 (4 studies)	no serious risk of bias	very serious[3,4]	very serious[1]	no serious imprecision[2]	undetected	⊕⊖⊖⊖ **VERY LOW**[1,2,3,4] due to inconsistency, indirectness	195 195	-	The mean 7 vas-r - combined intervention in the intervention groups was **0.9 lower** (1.9 lower to 0.1 higher)

1 Most (2/3) of the information comes from studies rated as high-risk.
2 The difference lies in the magnitude of the effect size (significant heterogeneity but consistent direction), which does not affect the conclusion and may be considered not to warrant downgrading.
3 The Optimal Information Size (OIS) was not met, as the total sample size was below the required threshold (300 for categoric al variables; 400 for continuous variables).
4 The confidence interval (CI) crossed the null line (0 for continuous outcomes, 1 for dichotomous outcomes).
5 All included RCTs were underpowered (n<30 pairs).

Bibliography: AART vs. Sham in TKA Rehab: VAS-R Comparison

Quality assessment							Summary of Findings				
**Participants (studies) Follow up**	**Risk of bias**	**Inconsistency**	**Indirectness**	**Imprecision**	**Publication bias**	**Overall quality of evidence**	**Study event rates (%)**		**Relative effect** (95% CI)	**Anticipated absolute effects**	
							**With shame AART on VAS-R scale**	**With AART**		**Risk with shame AART on VAS-R scale**	**Risk difference with AART** (95% CI)
**1VAS-R** (IMPORTANT OUTCOME; Better indicated by lower values)											
222 (3 studies)	no serious risk of bias	very serious[1,2]	no serious indirectness	serious[3,4]	undetected	⊕⊖⊖⊖ **VERY LOW**[1,2,3,4] due to inconsistency, imprecision	111	111	-		The mean 1vas-r in the intervention groups was **0.25 lower** (0 to 0.23 higher)
**1VAS-R - AP** (IMPORTANT OUTCOME; Better indicated by lower values)											
162 (2 studies)	no serious risk of bias	very serious[1,2]	no serious indirectness	no serious imprecision	undetected	⊕⊕⊖⊖ **LOW**[1,2] due to inconsistency	81	81	-		The mean 1vas-r - ap in the intervention groups was **0.03 lower** (0.11 lower to 0.05 higher)
**1VAS-R - AIDN** (Better indicated by lower values)											
60 (1 study)	no serious risk of bias	serious[1]	no serious indirectness	no serious imprecision	undetected	⊕⊕⊕⊖ **MODERATE**[1] due to inconsistency	30	30	-		The mean 1vas-r - aidn in the intervention groups was **0.8 lower** (1.43 to 0.17 lower)
**3VAS-R - AP** (Better indicated by lower values)											
162 (2 studies)	no serious risk of bias	serious[1]	no serious indirectness	no serious imprecision	undetected	⊕⊕⊕⊖ **MODERATE**[1] due to inconsistency	81	81	-		The mean 3vas-r - ap in the intervention groups was **0.31 lower** (0.42 to 0.21 lower)

1 The Optimal Information Size (OIS) was not met, as the total sample size was below the required threshold (300 for categoric al variables; 400 for continuous variables).
2 The confidence interval (CI) crossed the null line (0 for continuous outcomes, 1 for dichotomous outcomes).
3 The difference lies in the magnitude of the effect size (significant heterogeneity but consistent direction), which does not affect the conclusion and may be considered not to warrant downgrading.
4 The I² statistic was between 50% and 75%, indicating moderate to substantial heterogeneity.

Bibliography: AART vs. RRT in TKA Rehab: VAS-M Comparison

Quality assessment							Summary of Findings				
**Participants (studies) Follow up**	**Risk of bias**	**Inconsistency**	**Indirectness**	**Imprecision**	**Publication bias**	**Overall quality of evidence**	**Study event rates (%)**		**Relative effect** (95% CI)	**Anticipated absolute effects**	
							**With RRT on VAS-M scale**	**With AART**		**Risk with RRT on VAS-M scale**	**Risk difference with AART** (95% CI)
**1VAS-M** (Better indicated by lower values)											
254 (3 studies)	no serious risk of bias	very serious[1,2]	no serious indirectness	very serious[2,3]	undetected	⊕⊖⊖⊖ **VERY LOW**[1,2,3] due to inconsistency, imprecision	126	128	-		The mean 1vas-m in the intervention groups was **0.51 lower**

											(1.29 lower to 0.28 higher)
**1VAS-M - AP** (Better indicated by lower values)											
158 (2 studies)	no serious risk of bias	very serious[1,2]	no serious indirectness	very serious[3]	undetected	⊕⊝⊝⊝ **VERY LOW**[1,2,3] due to inconsistency, imprecision	78	80	-		The mean 1vas-m - ap in the intervention groups was **0.63 lower** (1.98 lower to 0.72 higher)
**1VAS-M - AIDN** (Better indicated by lower values)											
96 (1 study)	no serious risk of bias	very serious[1,2]	no serious indirectness	very serious[3]	undetected	⊕⊝⊝⊝ **VERY LOW**[1,2,3] due to inconsistency, imprecision	48	48	-		The mean 1vas-m - aidn in the intervention groups was **0.26 lower** (0.67 lower to 0.15 higher)
**3VAS-M** (Better indicated by lower values)											
254 (3 studies)	no serious risk of bias	serious[2]	no serious indirectness	no serious imprecision[4]	undetected	⊕⊕⊕⊝ **MODERATE**[2,4] due to inconsistency	126	128	-		The mean 3vas-m in the intervention groups was **0.74 lower** (1.16 to 0.32 lower)
**3VAS-M - AP** (Better indicated by lower values)											
158 (2 studies)	no serious risk of bias	serious[2]	no serious indirectness	no serious imprecision	undetected	⊕⊕⊕⊝ **MODERATE**[2] due to inconsistency	78	80	-		The mean 3vas-m - ap in the intervention groups was **0.52 lower** (0.85 to 0.19 lower)
**3VAS-M - AIDN** (Better indicated by lower values)											
96 (1 study)	no serious risk of bias	serious[2]	no serious indirectness	no serious imprecision	undetected	⊕⊕⊕⊝ **MODERATE**[2] due to inconsistency	48	48	-		The mean 3vas-m - aidn in the intervention groups was **1.09 lower** (1.54 to 0.64 lower)
**7VAS-M** (Better indicated by lower values)											
186 (2 studies)	no serious risk of bias	serious[2]	no serious indirectness	no serious imprecision[4]	undetected	⊕⊕⊕⊝ **MODERATE**[2,4] due to inconsistency	93	93	-		The mean 7vas-m in the intervention groups was **0.33 lower** (0.57 to 0.09 lower)

1 The confidence interval (CI) crossed the null line (0 for continuous outcomes, 1 for dichotomous outcomes).
2 The Optimal Information Size (OIS) was not met, as the total sample size was below the required threshold (300 for categoric al variables; 400 for continuous variables).
3 Substantial heterogeneity was observed (I² = 75%-100%).
4 The difference lies in the magnitude of the effect size (significant heterogeneity but consistent direction), which does not affect the conclusion and may be considered not to warrant

Bibliography: AART vs. RRT in TKA Rehab: HSS

Quality assessment							Summary of Findings				
**Participants (studies) Follow up**	**Risk of bias**	**Inconsistency**	**Indirectness**	**Imprecision**	**Publication bias**	**Overall quality of evidence**	**Study event rates (%)**		**Relative effect** (95% CI)	**Anticipated absolute effects**	
							**With Control**	**With HSS scale**		**Risk with Control**	**Risk difference with HSS**

										**scale** (95% CI)
**1HSS** (IMPORTANT OUTCOME; Better indicated by lower values)										
166 (2 studies)	no serious risk of bias	very serious[1,2]	very serious[3]	no serious imprecision	undetected	⊕⊖⊖⊖ **VERY LOW**[1,2,3] due to inconsistency, indirectness	83	83	-	The mean 1hss in the intervention groups was **0.34 higher** (0.79 lower to 1.47 higher)
**3HSS** (IMPORTANT OUTCOME; Better indicated by lower values)										
172 (2 studies)	no serious risk of bias	serious[2]	very serious[3]	no serious imprecision[4]	undetected	⊕⊖⊖⊖ **VERY LOW**[2,3,4] due to inconsistency, indirectness	86	86	-	The mean 3hss in the intervention groups was **4 higher** (1.32 to 6.68 higher)
**7HSS** (IMPORTANT OUTCOME; Better indicated by lower values)										
404 (4 studies)	no serious risk of bias	no serious inconsistency	very serious[3]	no serious imprecision[4]	undetected	⊕⊕⊖⊖ **LOW**[3,4] due to indirectness	202	202	-	The mean 7hss in the intervention groups was **5.55 higher** (1.68 to 9.41 higher)
**7HSS - AART vs. RRT** (IMPORTANT OUTCOME; Better indicated by lower values)										
304 (3 studies)	no serious risk of bias	serious[2]	very serious[3]	no serious imprecision[4]	undetected	⊕⊖⊖⊖ **VERY LOW**[2,3,4] due to inconsistency, indirectness	152	152	-	The mean 7hss - aart vs. rrt in the intervention groups was **5.76 higher** (0.19 to 11.33 higher)
**7HSS - AART vs. Sham** (IMPORTANT OUTCOME; Better indicated by lower values)										
100 (1 study)	no serious risk of bias	serious[2]	very serious[3]	no serious imprecision	undetected	⊕⊖⊖⊖ **VERY LOW**[2,3] due to inconsistency, indirectness	50	50	-	The mean 7hss - aart vs. sham in the intervention groups was **5 higher** (2.45 to 7.55 higher)
**14HSS** (IMPORTANT OUTCOME; Better indicated by lower values)										
150 (2 studies)	no serious risk of bias	serious[2]	serious[5]	no serious imprecision	undetected	⊕⊕⊖⊖ **LOW**[2,5] due to inconsistency, indirectness	75	75	-	The mean 14hss in the intervention groups was **3.74 higher** (1.27 lower to 8.76 higher)
**30HSS** (IMPORTANT OUTCOME; Better indicated by lower values)										
186 (2 studies)	no serious risk of bias	serious[2]	very serious[3]	no serious imprecision[4]	undetected	⊕⊖⊖⊖ **VERY LOW**[2,3,4] due to inconsistency, indirectness	93	93	-	The mean 30hss in the intervention groups was **11.25 higher** (5.19 to 17.32 higher)
**90HSS** (IMPORTANT OUTCOME; Better indicated by lower values)										
190 (2 studies)	no serious risk of bias	serious[2]	serious[5]	no serious imprecision[4]	undetected	⊕⊕⊖⊖ **LOW**[2,4,5] due to inconsistency,	95	95	-	The mean 90hss in the intervention

						indirectness				groups was **8.22 higher** (6.45 to 9.98 higher)

1 The confidence interval (CI) crossed the null line (0 for continuous outcomes, 1 for dichotomous outcomes).
2 The Optimal Information Size (OIS) was not met, as the total sample size was below the required threshold (300 for categoric al variables; 400 for continuous variables).
3 Most (2/3) of the information comes from studies rated as high-risk.
4 The difference lies in the magnitude of the effect size (significant heterogeneity but consistent direction), which does not affect the conclusion and may be considered not to warrant downgrading.
5 Most (2/3) of the information comes from studies rated as moderate-risk.

Bibliography: AART vs. RRT in TKA Rehab: Painkiller

Quality assessment							Summary of Findings				
**Participants (studies) Follow up**	**Risk of bias**	**Inconsistency**	**Indirectness**	**Imprecision**	**Publication bias**	**Overall quality of evidence**	**Study event rates (%)**		**Relative effect** (95% CI)	**Anticipated absolute effects**	
							**With Control**	**With Pain killer**		**Risk with Control**	**Risk difference with Pain killer** (95% CI)
**Step 3 opioids** (IMPORTANT OUTCOME; Better indicated by lower values)											
152 (3 studies)	no serious risk of bias	very serious[1,2]	no serious indirectness	no serious imprecision	undetected	⊕⊕⊖⊖ **LOW**[1,2] due to inconsistency	76	76	-		The mean step 3 opioids in the intervention groups was **7.16 lower** (16.49 lower to 2.17 higher)
**Step 2 opioids** (IMPORTANT OUTCOME)											
100 (2 studies)	no serious risk of bias	serious[2]	very serious[3]	no serious imprecision	undetected	⊕⊖⊖⊖ **VERY LOW**[2,3] due to inconsistency, indirectness	18/50 (36%)	3/50 (6%)	**OR 0.11** (0.03 to 0.42)	**Study population**	
										**360 per 1000**	**302 fewer per 1000** (from 169 fewer to 343 fewer)
										**Moderate**	
										**358 per 1000**	**300 fewer per 1000** (from 168 fewer to 342 fewer)
**PCA-POD2** (IMPORTANT OUTCOME; Better indicated by lower values)											
218 (3 studies)	no serious risk of bias	serious[2]	very serious[3]	no serious imprecision	undetected	⊕⊖⊖⊖ **VERY LOW**[2,3] due to inconsistency, indirectness	108	110	-		The mean pca-pod2 in the intervention groups was **22.68 lower** (47.46 lower to 2.09 higher)

1 The confidence interval (CI) crossed the null line (0 for continuous outcomes, 1 for dichotomous outcomes).
2 The Optimal Information Size (OIS) was not met, as the total sample size was below the required threshold (300 for categoric al variables; 400 for continuous variables).
3 Most (2/3) of the information comes from studies rated as high-risk.

Bibliography:AART vs. RRT in TKA Rehab: AROM

Quality assessment							Summary of Findings				
**Participants (studies) Follow up**	**Risk of bias**	**Inconsistency**	**Indirectness**	**Imprecision**	**Publication bias**	**Overall quality of evidence**	**Study event rates (%)**		**Relative effect** (95% CI)	**Anticipated absolute effects**	
							**With Control**	**With AROM**		**Risk with Control**	**Risk difference with AROM** (95% CI)

**7AROM** (IMPORTANT OUTCOME; Better indicated by lower values)											
326 (4 studies)	no serious risk of bias	serious[1]	very serious[2]	no serious imprecision[3]	undetected	⊕⊝⊝⊝ **VERY LOW**[1,2,3] due to inconsistency, indirectness	162	164	-		The mean 7arom in the intervention groups was **5.96 higher** (2.14 to 9.78 higher)
**14AROM** (Better indicated by lower values)											
300 (4 studies)	no serious risk of bias	serious[1]	very serious[2]	no serious imprecision[3]	undetected	⊕⊝⊝⊝ **VERY LOW**[1,2,3] due to inconsistency, indirectness	150	150	-		The mean 14arom in the intervention groups was **4.5 higher** (2.51 to 6.5 higher)

1 The confidence interval (CI) crossed the null line (0 for continuous outcomes, 1 for dichotomous outcomes).
2 Most (2/3) of the information comes from studies rated as high-risk.
3 The difference lies in the magnitude of the effect size (significant heterogeneity but consistent direction), which does not affect the conclusion and may be considered not to warrant downgrading.

Bibliography:AART vs. RRT in TKA Rehab: PRAEs

Quality assessment							Summary of Findings				
**Participants (studies) Follow up**	**Risk of bias**	**Inconsistency**	**Indirectness**	**Imprecision**	**Publication bias**	**Overall quality of evidence**	**Study event rates (%)**		**Relative effect** (95% CI)	**Anticipated absolute effects**	
							**With Control**	**With PRAEs**		**Risk with Control**	**Risk difference with PRAEs** (95% CI)
**Postoperative Vomiting** (IMPORTANT OUTCOME)											
572 (8 studies)	no serious risk of bias	no serious inconsistency	serious[1]	no serious imprecision	undetected	⊕⊕⊕⊝ **MODERATE**[1] due to indirectness	94/286 (32.9%)	26/286 (9.1%)	**OR 0.18** (0.11 to 0.29)	**Study population**	
										**329 per 1000**	**248 fewer per 1000** (from 204 fewer to 278 fewer)
										**Moderate**	
										**333 per 1000**	**251 fewer per 1000** (from 207 fewer to 281 fewer)
**Pruritus** (IMPORTANT OUTCOME)											
210 (3 studies)	no serious risk of bias	serious[2]	very serious[1]	no serious imprecision	undetected	⊕⊝⊝⊝ **VERY LOW**[1,2] due to inconsistency, indirectness	12/105 (11.4%)	4/105 (3.8%)	**OR 0.31** (0.1 to 0.99)	**Study population**	
										**114 per 1000**	**76 fewer per 1000** (from 1 fewer to 102 fewer)
										**Moderate**	
										**111 per 1000**	**74 fewer per 1000**

											(from 1 fewer to 99 fewer)
**DVT(Deep Vein Thrombosis)** (IMPORTANT OUTCOME)											
314 (3 studies)	no serious risk of bias	no serious inconsistency	very serious[1]	no serious imprecision	undetected	⊕⊕⊖⊖ **LOW**[1] due to indirectness	14/157 (8.9%)	3/157 (1.9%)	**OR 0.22** (0.07 to 0.73)	**Study population**	
										**89 per 1000**	**68 fewer per 1000** (from 22 fewer to 82 fewer)
										**Moderate**	
										**71 per 1000**	**54 fewer per 1000** (from 18 fewer to 66 fewer)
**Dizziness** (IMPORTANT OUTCOME)											
280 (4 studies)	no serious risk of bias	serious[2]	very serious[1]	no serious imprecision	undetected	⊕⊖⊖⊖ **VERY LOW**[1,2] due to inconsistency, indirectness	24/140 (17.1%)	7/140 (5%)	**OR 0.25** (0.11 to 0.61)	**Study population**	
										**171 per 1000**	**122 fewer per 1000** (from 59 fewer to 149 fewer)
										**Moderate**	
										**183 per 1000**	**130 fewer per 1000** (from 63 fewer to 159 fewer)
**Urine Retention** (IMPORTANT OUTCOME)											
300 (4 studies)	no serious risk of bias	no serious inconsistency	serious[3]	no serious imprecision	undetected	⊕⊕⊕⊖ **MODERATE**[3] due to indirectness	25/150 (16.7%)	6/150 (4%)	**OR 0.21** (0.08 to 0.52)	**Study population**	
										**167 per 1000**	**126 fewer per 1000** (from 72 fewer to 151 fewer)
										**Moderate**	
										**200 per 1000**	**150 fewer per 1000** (from 85 fewer to 180 fewer)
**Hematoma** (IMPORTANT OUTCOME)											
178 (2 studies)	no serious risk of bias	very serious[2,4]	very serious[1]	no serious imprecision	undetected	⊕⊖⊖⊖ **VERY LOW**[1,2,4] due to inconsistency, indirectness	4/89 (4.5%)	2/89 (2.2%)	**OR 0.49** (0.09 to 2.74)	**Study population**	
										**45 per 1000**	**22 fewer per 1000** (from 41 fewer to

										69 more)
									**Moderate**	
									**42 per 1000**	**21 fewer per 1000** (from 38 fewer to 65 more)

1 Most (2/3) of the information comes from studies rated as high-risk.
2 The Optimal Information Size (OIS) was not met, as the total sample size was below the required threshold (300 for categoric al variables; 400 for continuous variables).
3 Most (2/3) of the information comes from studies rated as moderate-risk.
4 The confidence interval (CI) crossed the null line (0 for continuous outcomes, 1 for dichotomous outcomes).
