# Supplementary material for: Does Auricular Acupoint‐Related Therapy Enhance Rehabilitation After Total Knee Arthroplasty? A Systematic Review and Network Meta‐Analysis
Source: Pain Res Manag. 2026 Sep 20;2026:9277186. doi: 10.1155/prm/9277186 (PMC13591118; doi:10.1155/prm/9277186)
Supplement: Supplementary file 2 [file PRM-2026-9277186-s001.docx]

Supplementary materials-1：

**Does Auricular acupoint-related therapy Enhance: Rehabilitation After Total Knee Arthroplasty? A Systematic Review and Network Meta-Analysis**

[1.Search strategy	3](#_Toc924575622)

[（1）PUBMED: 6	3](#_Toc150809262)

[（2）WOS: 11	3](#_Toc620562974)

[（3）EMBASE: 23	3](#_Toc1621314186)

[（4）Cochrane：20	4](#_Toc7527319)

[（5）CNKI: 40	5](#_Toc1957598907)

[（6）VIP: 63	5](#_Toc1915357909)

[（7）Wangfang: 61	6](#_Toc640508033)

[（8）Sinomed：39	6](#_Toc1830471867)

[2. Overview of complementary therapy interventions	8](#_Toc2037425394)

[3. Risk of bias assessment	9](#_Toc1381845543)

[Figure S1 Detailed Risk of Bias Assessment for Individual Studies	9](#_Toc1789882543)

[4. Forest plot and funnel plot for supplementary analyses.	10](#_Toc604973025)

[(1) Figure S2. Funnel Plot for Studies Reporting HSS Score	10](#_Toc1594046277)

[(2) Figure S3. Forest Plots for AART vs. RRT on HSS Score After TKA	11](#_Toc1277281214)

[(3) Figure S4. Funnel Plot for AART vs. RRT on Painkiller/ AROM After TKA	12](#_Toc1018828286)

[(4) Figure S5. Forest Plots for AART vs. RRT on Painkiller/ AROM After TKA	13](#_Toc1559885271)

[(5) Figure S6. Funnel Plot for AART vs. RRT on PRAEs After TKA	14](#_Toc511387121)

[(6) Figure S7. Forest Plots for AART vs. RRT on PRAEs After TKA	15](#_Toc653787353)

[5. SUCRA Curves	16](#_Toc1677703819)

[Figure S8. SUCRA Curves for Different Outcomes	16](#_Toc707800823)

# 1.Search strategy

## （1）PUBMED: 6

(( ("Arthroplasty, Replacement, Knee"[Mesh]) OR ("Arthroplasty, Replacement, Knee"[Title/Abstract] OR "Arthroplasty, Knee Replacement"[Title/Abstract] OR "Knee Replacement Arthroplasty"[Title/Abstract] OR "Total Knee Replacement"[Title/Abstract] OR "Knee Replacement, Total"[Title/Abstract] OR "Knee Arthroplasty"[Title/Abstract] OR "Arthroplasty, Knee"[Title/Abstract] OR "Knee Arthroplasty, Total"[Title/Abstract] OR "Arthroplasty, Total Knee"[Title/Abstract] OR "Total Knee Arthroplasty"[Title/Abstract]) )) AND (( ("Acupuncture, Ear"[Mesh]) OR ("Acupuncture, Ear"[Title/Abstract] OR "Ear Acupuncture"[Title/Abstract] OR "Auricular Acupuncture"[Title/Abstract] OR "Acupuncture, Auricular"[Title/Abstract] OR "Battlefield Acupuncture"[Title/Abstract] OR "Auriculotherapy"[Title/Abstract] OR "Auricular Therapy"[Title/Abstract] OR "Auricular Point Sticking"[Title/Abstract] OR "Auricular Point Pressing"[Title/Abstract] OR "Transcutaneous Auricular Vagus Nerve Stimulation"[Title/Abstract] OR "ta-VNS"[Title/Abstract] OR "Acupressure"[Title/Abstract] OR "Auricular Acupressure"[Title/Abstract]) )) Filters: Randomized Controlled Trial

## （2）WOS: 11

((TS=( Arthroplasty, Replacement, Knee) OR AB=( Arthroplasty, Replacement, Knee OR Arthroplasties, Replacement, Knee OR Arthroplasty, Knee Replacement OR Knee Replacement Arthroplasties OR Knee Replacement Arthroplasty OR Replacement Arthroplasties, Knee OR Replacement Arthroplasty, Knee OR Replacement, Total Knee OR Total Knee Replacement OR Knee Replacement, Total OR Knee Arthroplasty OR Arthroplasty, Knee OR Arthroplasties, Knee Replacement OR Knee Arthroplasty, Total OR Arthroplasty, Total Knee OR Total Knee Arthroplasty OR Arthroplasty, Replacement)) AND (TS=(Acupuncture, Ear) OR AB=(Acupunctures, Ear OR Ear Acupunctures OR Auricular Acupuncture OR Ear Acupuncture OR Acupuncture, Auricular OR Acupunctures, Auricular OR Auricular Acupunctures OR Battlefield Acupuncture OR Auriculotherapy OR Auricular Therapy OR Ear Massage OR Auricular Point Sticking OR Auricular Point Pressing OR Ears Pressure Beans OR Transcutaneous Auricular Vagus Nerve Stimulation OR ta-VNS OR Ear Point Moxibustion OR Ear Point Bloodletting OR Ear Point Injection OR Acupressure OR Auricular acupressure)) AND (TS=(Randomized Controlled Trial) OR AB=(Randomized Controlled Trial OR Randomized OR Random Allocation OR Clinical Trials, Randomized OR Trials, Randomized Clinical OR Controlled Clinical Trials, Randomized OR RCT)))

## （3）EMBASE: 23

('knee replacement'/exp OR 'knee replacement' OR 'knee arthroplasty'/exp OR 'knee arthroplasty' OR 'unicompartmental knee arthroplasty'/exp OR 'unicompartmental knee arthroplasty' OR 'partial knee replacement'/exp OR 'partial knee replacement' OR 'arthroplasty, replacement, knee':ti,ab OR 'arthroplasties, replacement, knee':ti,ab OR 'arthroplasty, knee replacement':ti,ab OR 'knee replacement arthroplasties':ti,ab OR 'knee replacement arthroplasty':ti,ab OR 'replacement arthroplasties, knee':ti,ab OR 'replacement arthroplasty, knee':ti,ab OR 'replacement, total knee':ti,ab OR 'total knee replacement':ti,ab OR 'knee replacement, total':ti,ab OR 'knee arthroplasty':ti,ab OR 'arthroplasty, knee':ti,ab OR 'arthroplasties, knee replacement':ti,ab OR 'knee arthroplasty, total':ti,ab OR 'arthroplasty, total knee':ti,ab OR 'total knee arthroplasty':ti,ab) AND ('ear acupuncture'/exp OR 'ear acupuncture' OR 'auriculotherapy'/exp OR 'auriculotherapy' OR 'acupressure'/exp OR 'acupressure' OR 'transcutaneous vagus nerve stimulation'/exp OR 'transcutaneous vagus nerve stimulation' OR 'acupuncture, ear':ti,ab OR 'acupunctures, ear':ti,ab OR 'ear acupunctures':ti,ab OR 'auricular acupuncture':ti,ab OR 'ear acupuncture':ti,ab OR 'acupuncture, auricular':ti,ab OR 'acupunctures, auricular':ti,ab OR 'auricular acupunctures':ti,ab OR 'battlefield acupuncture':ti,ab OR 'auriculotherapy':ti,ab OR 'auricular therapy':ti,ab OR 'ear massage':ti,ab OR 'auricular point sticking':ti,ab OR 'auricular point pressing':ti,ab OR 'ears pressure beans':ti,ab OR 'transcutaneous auricular vagus nerve stimulation':ti,ab OR 'ta-vns':ti,ab OR 'ear point moxibustion':ti,ab OR 'ear point bloodletting':ti,ab OR 'ear point injection':ti,ab OR 'acupressure':ti,ab OR 'auricular acupressure':ti,ab) AND ('randomized controlled trial'/exp OR 'randomized controlled trial' OR 'randomization'/exp OR 'randomization' OR 'randomized controlled trial':ti,ab OR 'randomized':ti,ab OR 'random allocation':ti,ab OR 'clinical trials, randomized':ti,ab OR 'trials, randomized clinical':ti,ab OR 'controlled clinical trials, randomized':ti,ab OR 'rct':ti,ab)

## （4）Cochrane：20

(Arthroplasty, Replacement, Knee OR Arthroplasties, Replacement, Knee OR Arthroplasty, Knee Replacement OR Knee Replacement Arthroplasties OR Knee Replacement Arthroplasty OR Replacement Arthroplasties, Knee OR Replacement Arthroplasty, Knee OR Replacement, Total Knee OR Total Knee Replacement OR Knee Replacement, Total OR Knee Arthroplasty OR Arthroplasty, Knee OR Arthroplasties, Knee Replacement OR Knee Arthroplasty, Total OR Arthroplasty, Total Knee OR Total Knee Arthroplasty OR Arthroplasty, Replacement, Partial Knee OR Unicompartmental Knee Arthroplasty OR Arthroplasty, Unicompartmental Knee OR Knee Arthroplasty, Unicompartmental OR Unicompartmental Knee Replacement OR Knee Replacement, Unicompartmental OR Partial Knee Replacement OR Knee Replacement, Partial OR Unicondylar Knee Replacement OR Knee Replacement, Unicondylar OR Unicondylar Knee Arthroplasty OR Arthroplasty, Unicondylar Knee OR Knee Arthroplasty, Unicondylar OR Partial Knee Arthroplasty OR Arthroplasty, Partial Knee OR Knee Arthroplasty, Partial):ti,ab,kw AND (Acupunctures, Ear OR Ear Acupunctures OR Auricular Acupuncture OR Ear Acupuncture OR Acupuncture, Auricular OR Acupunctures, Auricular OR Auricular Acupunctures OR Battlefield Acupuncture OR Auriculotherapy OR Auricular Therapy OR Ear Massage OR Auricular Point Sticking OR Auricular Point Pressing OR Ears Pressure Beans OR Transcutaneous Auricular Vagus Nerve Stimulation OR ta-VNS OR Ear Point Moxibustion OR Ear Point Bloodletting OR Ear Point Injection OR Acupressure OR Auricular acupressure):ti,ab,kw AND (Randomized Controlled Trial OR Randomized OR Random Allocation OR Clinical Trials, Randomized OR Trials, Randomized Clinical OR Controlled Clinical Trials, Randomized OR RCT):ti,ab,kw

## （5）CNKI: 40

## （6）VIP: 63

((T=关节成形术,置换,膝 OR T=膝置换关节成形术 OR T=全膝关节置换术 OR T=全膝关节成形术 OR T=全膝关节置换成形术 OR T=膝关节置换 OR T=膝置换术 OR T=TKA OR T=TKR) OR (K=关节成形术,置换,膝 OR K=膝置换关节成形术 OR K=全膝关节置换术 OR K=全膝关节成形术 OR K=全膝关节置换成形术 OR K=膝关节置换 OR K=膝置换术 OR K=TKA OR K=TKR) OR (R=关节成形术,置换,膝 OR R=膝置换关节成形术 OR R=全膝关节置换术 OR R=全膝关节成形术 OR R=全膝关节置换成形术 OR R=膝关节置换 OR R=膝置换术 OR R=TKA OR R=TKR)) AND ((T=耳穴 OR T=耳穴贴压 OR T=耳针疗法 OR T=耳穴疗法 OR T=耳豆贴压 OR T=耳穴压豆 OR T=耳豆 OR T=耳甲迷走神经 OR T=耳皮迷走神经 OR T=经皮耳穴迷走神经 OR T=耳灸 OR T=耳穴放血 OR T=耳穴按摩 OR T=耳穴注射 OR T=熨耳法 OR T=耳穴刮痧) OR (K=耳穴 OR K=耳穴贴压 OR K=耳针疗法 OR K=耳穴疗法 OR K=耳豆贴压 OR K=耳穴压豆 OR K=耳豆 OR K=耳甲迷走神经 OR K=耳皮迷走神经 OR K=经皮耳穴迷走神经 OR K=耳灸 OR K=耳穴放血 OR K=耳穴按摩 OR K=耳穴注射 OR K=熨耳法 OR K=耳穴刮痧) OR (R=耳穴 OR R=耳穴贴压 OR R=耳针疗法 OR R=耳穴疗法 OR R=耳豆贴压 OR R=耳穴压豆 OR R=耳豆 OR R=耳甲迷走神经 OR R=耳皮迷走神经 OR R=经皮耳穴迷走神经 OR R=耳灸 OR R=耳穴放血 OR R=耳穴按摩 OR R=耳穴注射 OR R=熨耳法 OR R=耳穴刮痧)) AND ((T=随机对照研究 OR T=随机对照试验 OR T=随机 OR T=随机分配 OR T=RCT OR T=随机化 OR T=随机化分配) OR (K=随机对照研究 OR K=随机对照试验 OR K=随机 OR K=随机分配 OR K=RCT OR K=随机化 OR K=随机化分配) OR (R=随机对照研究 OR R=随机对照试验 OR R=随机 OR R=随机分配 OR R=RCT OR R=随机化 OR R=随机化分配))

## （7）Wangfang: 61

(主题:("关节成形术 置换 膝" OR "全膝关节置换术" ) OR 题名或关键词:("关节成形术 置换 膝" OR "全膝关节置换术") OR 摘要:("关节成形术 置换 膝" OR "膝置换关节成形术" OR "全膝关节置换术" OR "全膝关节成形术" OR "全膝关节置换成形术" OR "TKA" OR "TKR")) AND (主题:("耳穴贴压" OR "耳针疗法" OR "耳穴疗法") OR 题名或关键词:("耳穴贴压" OR "耳针疗法" OR "耳穴疗法") OR 摘要:("耳穴" OR "耳穴贴压" OR "耳针疗法" OR "耳穴疗法" OR "耳豆贴压" OR "耳穴压豆" OR "耳豆" OR "耳甲迷走神经" OR "耳皮迷走神经" OR "经皮耳穴迷走神经" OR "耳灸" OR "耳穴放血" OR "耳穴按摩" OR "耳穴注射" OR "熨耳法" OR "耳穴刮痧" OR "耳针")) AND (主题:("随机对照试验" OR "RCT" OR "随机对照研究") OR 题名或关键词:("随机对照试验" OR "RCT" OR "随机对照研究") OR 摘要:("随机对照研究" OR "随机对照试验" OR "随机" OR "随机分配" OR "RCT" OR "随机化" OR "随机化分配"))

## （8）Sinomed：39

序号	检索表达式	命中文献数	检索时间

1)	( "耳穴"[加权:扩展] OR "耳穴贴压"[加权:扩展] OR "耳针疗法"[加权:扩展] OR "耳穴疗法"[加权:扩展] OR "耳豆贴压"[加权:扩展] OR "耳穴压豆"[加权:扩展] OR "耳豆"[加权:扩展] OR "耳针疗法"[加权:扩展] OR "耳甲迷走神经"[加权:扩展] OR "耳皮迷走神经"[加权:扩展] OR "经皮耳穴迷走神经"[加权:扩展] OR "耳灸"[加权:扩展] OR "耳穴放血"[加权:扩展] OR "耳穴按摩"[加权:扩展] OR "耳穴注射"[加权:扩展] OR "熨耳法"[加权:扩展] OR "耳穴刮痧"[加权:扩展]) OR( "耳穴"[关键词:智能] OR "耳穴贴压"[关键词:智能] OR "耳针疗法"[关键词:智能] OR "耳穴疗法"[关键词:智能] OR "耳豆贴压"[关键词:智能] OR "耳穴压豆"[关键词:智能] OR "耳豆"[关键词:智能] OR "耳针疗法"[关键词:智能] OR "耳甲迷走神经"[关键词:智能] OR "耳皮迷走神经"[关键词:智能] OR "经皮耳穴迷走神经"[关键词:智能] OR "耳灸"[关键词:智能] OR "耳穴放血"[关键词:智能] OR "耳穴按摩"[关键词:智能] OR "耳穴注射"[关键词:智能] OR "熨耳法"[关键词:智能] OR "耳穴刮痧"[关键词:智能]) OR( "耳穴"[摘要:智能] OR "耳穴贴压"[摘要:智能] OR "耳针疗法"[摘要:智能] OR "耳穴疗法"[摘要:智能] OR "耳豆贴压"[摘要:智能] OR "耳穴压豆"[摘要:智能] OR "耳豆"[摘要:智能] OR "耳针疗法"[摘要:智能] OR "耳甲迷走神经"[摘要:智能] OR "耳皮迷走神经"[摘要:智能] OR "经皮耳穴迷走神经"[摘要:智能] OR "耳灸"[摘要:智能] OR "耳穴放血"[摘要:智能] OR "耳穴按摩"[摘要:智能] OR "耳穴注射"[摘要:智能] OR "熨耳法"[摘要:智能] OR "耳穴刮痧"[摘要:智能]) OR( "耳穴"[中文标题:智能] OR "耳穴贴压"[中文标题:智能] OR "耳针疗法"[中文标题:智能] OR "耳穴疗法"[中文标题:智能] OR "耳豆贴压"[中文标题:智能] OR "耳穴压豆"[中文标题:智能] OR "耳豆"[中文标题:智能] OR "耳针疗法"[中文标题:智能] OR "耳甲迷走神经"[中文标题:智能] OR "耳皮迷走神经"[中文标题:智能] OR "经皮耳穴迷走神经"[中文标题:智能] OR "耳灸"[中文标题:智能] OR "耳穴放血"[中文标题:智能] OR "耳穴按摩"[中文标题:智能] OR "耳穴注射"[中文标题:智能] OR "熨耳法"[中文标题:智能] OR "耳穴刮痧"[中文标题:智能])	16259	2026-06-30 17:28:07.0

3)	( "随机对照研究"[加权:扩展] OR "随机对照试验"[加权:扩展] OR "随机"[加权:扩展] OR "随机分配"[加权:扩展] OR "RCT"[加权:扩展] OR "随机化"[加权:扩展] OR "随机化分配"[加权:扩展]) OR( "随机对照研究"[关键词:智能] OR "随机对照试验"[关键词:智能] OR "随机"[关键词:智能] OR "随机分配"[关键词:智能] OR "RCT"[关键词:智能] OR "随机化"[关键词:智能] OR "随机化分配"[关键词:智能]) OR( "随机对照研究"[中文标题:智能] OR "随机对照试验"[中文标题:智能] OR "随机"[中文标题:智能] OR "随机分配"[中文标题:智能] OR "RCT"[中文标题:智能] OR "随机化"[中文标题:智能] OR "随机化分配"[中文标题:智能]) OR( "随机对照研究"[摘要:智能] OR "随机对照试验"[摘要:智能] OR "随机"[摘要:智能] OR "随机分配"[摘要:智能] OR "RCT"[摘要:智能] OR "随机化"[摘要:智能] OR "随机化分配"[摘要:智能])	2029727	2026-06-30 17:29:31.0

4)	( "关节成形术,置换,膝"[常用字段:智能] OR "膝置换关节成形术"[常用字段:智能] OR "全膝关节置换术"[常用字段:智能] OR "全膝关节成形术"[常用字段:智能] OR "全膝关节置换成形术"[常用字段:智能]) OR( "关节成形术,置换,膝"[常用字段:智能] OR "膝置换关节成形术"[常用字段:智能] OR "全膝关节置换术"[常用字段:智能] OR "全膝关节成形术"[常用字段:智能] OR "全膝关节置换成形术"[常用字段:智能]) OR( "关节成形术,置换,膝"[常用字段:智能] OR "膝置换关节成形术"[常用字段:智能] OR "全膝关节置换术"[常用字段:智能] OR "全膝关节成形术"[常用字段:智能] OR "全膝关节置换成形术"[常用字段:智能]) OR( "关节成形术,置换,膝"[常用字段:智能] OR "膝置换关节成形术"[常用字段:智能] OR "全膝关节置换术"[常用字段:智能] OR "全膝关节成形术"[常用字段:智能] OR "全膝关节置换成形术"[常用字段:智能])	11745	2026-06-30 17:32:17.0

5)	(#4) AND (#3) AND (#1)	37	2026-06-30 17:32:25.0

# 2. Overview of complementary therapy interventions

1. Auricular Acupressure (AP)

Auricular acupressure is a therapeutic technique in which small round pellets (e.g., Vaccaria seeds) are applied to the surface of specific auricular points. Patients or caregivers are typically instructed to apply intermittent manual pressure to these points for preventive or therapeutic purposes.

3. Auricular Intradermal Needling (AIDN)

Auricular intradermal needling involves the insertion of specially designed small-gauge intradermal needles into selected auricular points, where they remain fixed for a period of time. This method provides continuous stimulation to the acupoints for preventive or therapeutic purposes.

4. Chinese Medicine Transdermal Therapy (CMTT)

Chinese Medicine Transdermal Therapy refers to a therapeutic method in which Chinese herbal medicines in various dosage forms (e.g., pastes, ointments, gels, or liquid preparations) are applied to the skin surface. Through natural permeation of the active components or with the assistance of physical modalities such as pulsed current, the herbal constituents are delivered transdermally to exert local or systemic therapeutic effects.

5. Moxibustion (Mox)

Moxibustion is a thermal therapy in which compressed mugwort (Artemisia vulgaris) is burned near or on the skin to warm specific acupoints or areas, thereby preventing or treating disease.

6. Transcutaneous Auricular Vagus Nerve Stimulation (Ta-VNS)

Transcutaneous Auricular Vagus Nerve Stimulation is a non-invasive neuromodulation technique that delivers low-intensity electrical stimulation to the auricular branch of the vagus nerve via electrodes placed on the skin of the auricle. This method aims to modulate pain perception and autonomic function through activation of vagal pathways.

7. Body Acupuncture (BA)

Body acupuncture is a traditional needling technique involving the insertion of fine filiform needles into specific acupoints located on the body (non-auricular regions).

8. Auricular acupuncture (AA)

Auricular acupuncture, distinguished here from body acupuncture, refers to an invasive needling therapy that uses filiform needles inserted into specific points on the auricle to prevent and treat disease.

# 3. Risk of bias assessment

## Figure S1 Detailed Risk of Bias Assessment for Individual Studies

**Note:** In most studies, the "randomization process" was rated as having a "high risk" of bias, primarily because these studies only described the randomization process without mentioning allocation concealment or had obvious issues with allocation concealment. The dimension of "deviations from intended interventions" generally exhibited "some concerns" or "high risk," mainly due to difficulties in implementing blinding for interveners and patients in some AART studies. All studies did not report significant dropout or missing data, so the "missing outcome data" dimension was rated as low risk. In the "measurement of the outcome" dimension, reliance on patient-reported outcomes and subjective scales, or the lack of description of blinding for outcome assessors, raised concerns about measurement bias or led to a high-risk assessment. Most studies showed "some concerns" in the "selection of the reported result" dimension, commonly due to the absence of pre-registration, making it impossible to determine whether selective reporting existed.

**Supplementary Description:** Figure S1 provides a detailed summary of the risk of bias assessment for all included studies across multiple domains, including randomization, deviations from interventions, missing data, measurement of outcomes, and selection of reported results.

# 4. Forest plot and funnel plot for supplementary analyses.

## (1) Figure S2. Funnel Plot for Studies Reporting HSS Score

**Note:** A. AART vs. RRT for POD1 HSS. B. AART vs. RRT for POD3 HSS. C. AART vs. RRT for POD7 HSS. D. AART vs. RRT for POD14 HSS. E. AART vs. RRT for POD30 HSS. F. AART vs. RRT for POD90 HSS.

**Supplementary Description:** Figure S2 presents funnel plots for studies reporting HSS scores at different postoperative days (POD1, POD3, POD7, POD14, POD30, and POD90), which are used to assess potential publication bias.

## (2) Figure S3. Forest Plots for AART vs. RRT on HSS Score After TKA

**Note:** A. AART vs. RRT for POD1 HSS. B. AART vs. RRT for POD3 HSS. C. AART vs. RRT for POD7 HSS. D. AART vs. RRT for POD14 HSS. E. AART vs. RRT for POD30 HSS. F. AART vs. RRT for POD90 HSS.

**Supplementary Description:** Figure S3 displays forest plots comparing the effects of Auricular Acupoint-Related Therapy (AART) versus Routine Rehabilitation Therapy (RRT) on HSS scores at six postoperative time points (POD1, 3, 7, 14, 30, and 90), illustrating the effect sizes and confidence intervals for each comparison.

## (3) Figure S4. Funnel Plot for AART vs. RRT on Painkiller/ AROM After TKA

**Note:** A. AART vs. RRT for Step 2 opioids. B. AART vs. RRT for Step 3 opioids. C. AART vs. RRT for POD2 PCA. D. AART vs. RRT for POD7 AROM. E. AART vs. RRT for POD14 AROM.

**Supplementary Description:** Figure S4 presents funnel plots for studies evaluating painkiller usage (Step 2 opioids, Step 3 opioids, POD2 PCA) and active range of motion (AROM at POD7 and POD14), serving as a visual test for potential publication bias across these outcomes.

## (4) Figure S5. Forest Plots for AART vs. RRT on Painkiller/ AROM After TKA

**Note:** A. AART vs. RRT for Step 2 opioids. B. AART vs. RRT for Step 3 opioids. C. AART vs. RRT for POD2 PCA. D. AART vs. RRT for POD7 AROM. E. AART vs. RRT for POD14 AROM.

**Supplementary Description:** Figure S5 provides forest plots detailing the comparative effects of AART versus RRT on analgesic consumption (Step 2 opioids, Step 3 opioids, and PCA on POD2) and active range of motion (AROM at POD7 and POD14) following total knee arthroplasty.

## (5) Figure S6. Funnel Plot for AART vs. RRT on PRAEs After TKA

**Note:** A. AART vs. RRT for Postoperative Vomiting. B. AART vs. RRT for Pruritus. C. AART vs. RRT for DVT. D. AART vs. RRT for Dizziness. E. AART vs. RRT for Urine Retention. F. AART vs. RRT for Hematoma.

**Supplementary Description:** Figure S6 illustrates funnel plots for studies reporting postoperative adverse events (PRAEs), including vomiting, pruritus, deep vein thrombosis (DVT), dizziness, urine retention, and hematoma, used to assess publication bias in safety outcomes.

## (6) Figure S7. Forest Plots for AART vs. RRT on PRAEs After TKA

**Note:** A. AART vs. RRT for Postoperative Vomiting. B. AART vs. RRT for Pruritus. C. AART vs. RRT for DVT. D. AART vs. RRT for Dizziness. E. AART vs. RRT for Urine Retention. F. AART vs. RRT for Hematoma.

**Supplementary Description:** Figure S7 shows forest plots comparing the incidence of postoperative adverse events (PRAEs) between AART and RRT groups, including vomiting, pruritus, DVT, dizziness, urine retention, and hematoma, with pooled effect estimates for each complication.

# 5. SUCRA Curves

## Figure S8. SUCRA Curves for Different Outcomes

**Note:** A. SCURA Curves for POD1 VAS-R. B. SCURA Curves for POD3 VAS-R. C. SCURA Curves for POD7 VAS-R. D. SCURA Curves for POD7 AROM.

**Supplementary Description:** Figure S8 presents the Surface Under the Cumulative Ranking (SUCRA) curves for different interventions across multiple outcomes, including VAS-R scores at POD1, POD3, and POD7, as well as AROM at POD7, illustrating the probability ranking of each treatment's effectiveness.
